# Continuation of fluoropyrimidine treatment with S-1 after cardiotoxicity on capecitabine- or 5-fluorouracil-based therapy in patients with solid tumours: a multicentre retrospective observational cohort study☆

**DOI:** 10.1016/j.esmoop.2022.100427

**Published:** 2022-03-30

**Authors:** P. Osterlund, S. Kinos, P. Pfeiffer, T. Salminen, J.J.M. Kwakman, J.-E. Frödin, C.H. Shah, H. Sorbye, R. Ristamäki, P. Halonen, L.M. Soveri, E. Heervä, A. Ålgars, M. Bärlund, H. Hagman, R. McDermott, M. O’Reilly, R. Röckert, G. Liposits, R. Kallio, P. Flygare, A.J. Teske, E. van Werkhoven, C.J.A. Punt, B. Glimelius

**Affiliations:** 1 Department of Oncology, Tampere University Hospital and University of Tampere, Tampere, Finland; 2 Department of Oncology and Pathology, Karolinska Institutet and Karolinska University Hospital, Stockholm, Sweden; 3 Department of Oncology, Helsinki University Hospital and University of Helsinki, Helsinki, Finland; 4 Department of Oncology, Odense University Hospital, Odense, Denmark; 5 Department of Oncology, Amsterdam University Medical Centre, Amsterdam, the Netherlands; 6 Department of Oncology, Karolinska University Hospital, Stockholm, Sweden; 7 Department of Oncology, Haukeland University Hospital and Department of Clinical Science, University of Bergen, Bergen, Norway; 8 Department of Oncology, Turku University Hospital and University of Turku, Turku, Finland; 9 Department of Oncology, Skåne University Hospital, Lund, Sweden; 10 Department of Oncology, St. Vincent’s University Hospital, Dublin, Ireland; 11 Department of Oncology, Uppsala University Hospital, Uppsala, Sweden; 12 Department of Oncology, Regional Hospital West Jutland, Herning, Denmark; 13 Department of Oncology, Oulu University Hospital, Oulu, Finland; 14 Department of Oncology, Sundsvall Hospital, Sundsvall, Sweden; 15 Department of Cardiology, University Medical Centre Utrecht, Utrecht, the Netherlands; 16 Department of Biometrics, Netherlands Cancer Institute, Amsterdam, the Netherlands; 17 Department of Epidemiology, Julius Centre for Health Sciences and Primary Care, University Medical Centre Utrecht, Utrecht University, Utrecht, the Netherlands; 18 Department of Immunology, Genetics and Pathology, Uppsala University, Uppsala, Sweden

**Keywords:** cardiac toxicity, cardiotoxicity, fluoropyrimidines, S-1, gastrointestinal cancer, colorectal cancer, ACS, acute coronary syndrome, ATC, anatomical therapeutic chemical, CI, confidence interval, DPD, dihydroxypyrimidine dehydrogenase, ECG, electrocardiogram, FBAL, α-fluoro-β-alanine 5-FU, 5-fluorouracil, IQR, interquartile range, mCRC, metastatic colorectal cancer, MI, myocardial infarction, MINEN, mixed neuroendocrine non-neuroendocrine neoplasms, OR, odds ratio, OS, overall survival, PS, performance status

## Abstract

**Background:**

Capecitabine- or 5-fluorouracil (5-FU)-based chemotherapy is widely used in many solid tumours, but is associated with cardiotoxicity. S-1 is a fluoropyrimidine with low rates of cardiotoxicity, but evidence regarding the safety of switching to S-1 after 5-FU- or capecitabine-associated cardiotoxicity is scarce.

**Patients and methods:**

This retrospective study (NCT04260269) was conducted at 13 centres in 6 countries. The primary endpoint was recurrence of cardiotoxicity after switch to S-1-based treatment due to 5-FU- or capecitabine-related cardiotoxicity: clinically meaningful if the upper boundary of the 95% confidence interval (CI; by competing risk) is not including 15%. Secondary endpoints included cardiac risk factors, diagnostic work-up, treatments, outcomes, and timelines of cardiotoxicity.

**Results:**

Per protocol, 200 patients, treated between 2011 and 2020 [median age 66 years (range 19-86); 118 (59%) males], were included. Treatment intent was curative in 145 (73%). Initial cardiotoxicity was due to capecitabine (*n* = 170), continuous infusion 5-FU (*n* = 22), or bolus 5-FU (*n* = 8), which was administered in combination with other chemotherapy, targeted agents, or radiotherapy in 133 patients. Previous cardiovascular comorbidities were present in 99 (50%) patients. Cardiotoxic events (*n* = 228/200) included chest pain (*n* = 125), coronary syndrome/infarction (*n* = 69), arrhythmia (*n* = 22), heart failure/cardiomyopathy (*n* = 7), cardiac arrest (*n* = 4), and malignant hypertension (*n* = 1). Cardiotoxicity was severe or life-threatening in 112 (56%) patients and led to permanent capecitabine/5-FU discontinuation in 192 (96%). After switch to S-1, recurrent cardiotoxicity was observed in eight (4%) patients (95% CI 2.02-7.89, primary endpoint met). Events were limited to grade 1-2 and occurred at a median of 16 days (interquartile range 7-67) from therapy switch. Baseline ischemic heart disease was a risk factor for recurrent cardiotoxicity (odds ratio 6.18, 95% CI 1.36-28.11).

**Conclusion:**

Switching to S-1-based therapy is safe and feasible after development of cardiotoxicity on 5-FU- or capecitabine-based therapy and allows patients to continue their pivotal fluoropyrimidine-based treatment.

## Highlights

• Cardiotoxicity occurs in 4%-6% during capecitabine- or 5-FU-based treatment and often leads to treatment discontinuation.
• This is the largest patient series switched to S-1-based treatment after cardiotoxicity on fluoropyrimidine treatment.
• We show that 96% of 200 patients had no recurrent cardiotoxicity upon switch to S-1; grade 1-2 was seen in 4%.
• The essential fluoropyrimidine treatment, as S-1-based chemotherapy, was completed in 99% of patients.
• S-1 is feasible for life-prolonging or lifesaving treatment when capecitabine/5-FU is discontinued due to cardiotoxicity.

## Graphical abstract

**Figure:**
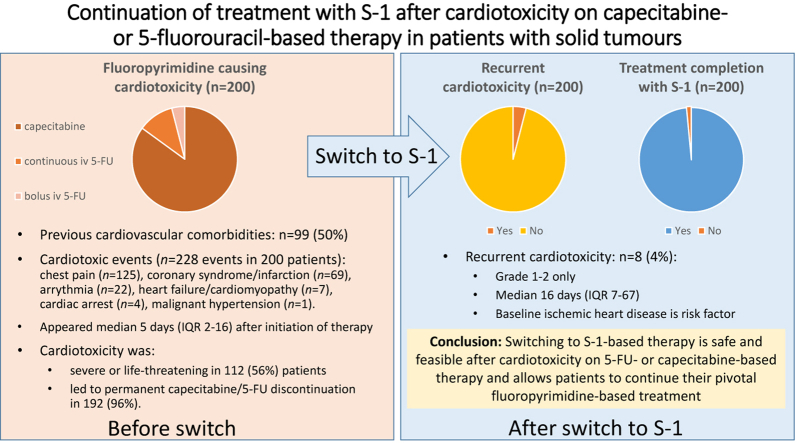

## Introduction

Fluoropyrimidines, including oral capecitabine and continuous infusion or bolus 5-fluorouracil (5-FU), are the cornerstone of curative or life-prolonging chemotherapy in many solid tumours.^1^^,^^2^ Cardiotoxicity is a common and potentially lethal complication of fluoropyrimidine treatment, with a reported incidence varying between 0% and 35%, depending on assessment method, dose, and schedule.^3^, ^4^, ^5^, ^6^ Cardiologist verified population- or trial-based reports demonstrate cardiotoxicity incidence rates of 4%-6% in patients receiving capecitabine or infused 5-FU.^6^, ^7^, ^8^

Angina-like chest pain, with or without ischemia, is the predominant clinical presentation, and frequently occurs early after fluoropyrimidine administration.^6^^,^^8^ Serious or life-threatening adverse events, including acute coronary syndrome, myocardial infarction, arrhythmias, heart failure, cardiogenic shock, and sudden death, have each been reported in 0.1%-4.6%.^6^, ^7^, ^8^ Most cardiotoxic events leading to permanent discontinuation occur within days from treatment initiation, with the consequence that these patients derive no benefit from this treatment.

Dose reduction and rechallenge lead to recurrence of cardiotoxicity in 44%-90% of patients, even with prophylactic calcium blocker or nitrate treatment.^4^^,^^6^^,^^9^, ^10^, ^11^ This illustrates the challenge of continuing potentially beneficial fluoropyrimidine treatment once cardiotoxicity has occurred.

The pathophysiological mechanisms underlying these cardiotoxic effects are unknown but probably multifactorial.^4^^,^^5^^,^^12^ Existing cardiac comorbidity has not reliably been shown to be a predisposing factor, and >50% have no previous coronary disease.^3^^,^^4^^,^^10^^,^^12^^,^^13^ Coronary vasospasm induced by 5-FU or its metabolites has been suggested as one possible mechanism.^4^^,^^12^ Indeed, most 5-FU (85%-90%) is rapidly catabolised by dihydroxypyrimidine dehydrogenase (DPD) to generate α-fluoro-β-alanine (FBAL), F-citrate, and fluoroacetate, all with direct toxic effects on the myocardium.^4^^,^^12^ Alternative fluoropyrimidines, such as S-1, that reduce the levels of these metabolites could be associated with lower cardiotoxicity rates.^12^ 5-FU can also induce reversible endothelial injury, with impaired vasodilation of vascular smooth muscle, leading to a procoagulant state.^4^^,^^14^

S-1 is widely used in Asian populations (details in Supplementary Table S1, available at https://doi.org/10.1016/j.esmoop.2022.100427), and is a combination of the fluoropyrimidine tegafur with two metabolic inhibitors designed to slow metabolism of 5-FU: gimeracil, a DPD inhibitor, and potassium oxonate, an inhibitor of the orotate phosphoribosyltransferase that converts 5-FU to fluorouridine monophosphate.^12^ In Western patients, comparable efficacy with an altered safety profile has been reported for S-1 compared with capecitabine in patients with metastatic colorectal cancer (mCRC)^15^ and to 5-FU in advanced gastric cancer.^16^ Also, a meta-analysis of both Asian and Western studies in oesophagogastric cancer reported similar efficacy to capecitabine or 5-FU, but different toxicity profiles, favouring S-1.^17^ Cardiotoxicity, however, was not reported in these studies. One case report of seven patients successfully switched to S-1 after cardiotoxicity on another fluoropyrimidine has suggested that this approach is safe, but further data are needed.^18^

This retrospective multicentre study aimed to evaluate the safety of S-1 after switch from another fluoropyrimidine due to cardiotoxicity and to identify possible risk factors for cardiotoxicity. A retrospective design was chosen as a prospective trial was not judged to be feasible due to long timeframes for expected inclusion and the consideration that randomization against re-exposure would be unethical.

## Methods

### Study design

This retrospective cohort study (Supplementary Material S1, available at https://doi.org/10.1016/j.esmoop.2022.100427) was conducted at 13 centres in Finland, Sweden, Norway, Denmark, The Netherlands, and Ireland (centres described in Supplementary Table S2, available at https://doi.org/10.1016/j.esmoop.2022.100427). The study was approved by each institution and/or the local ethics committee, if required. The study was conducted according to Good Clinical Practice guidelines and the Declaration of Helsinki, as applicable for register studies.

The primary endpoint was recurrence of cardiotoxicity after switch to S-1-based treatment from any other fluoropyrimidine due to cardiotoxicity. Secondary endpoints were cardiac symptoms and alterations in cardiac functional parameters during fluoropyrimidine therapy, diagnostic work-up for cardiotoxicity in real-world practice, timelines for cardiotoxicity, dose intensity, and outcomes.

Cardiac adverse events, i.e. cardiotoxicity, were defined and graded using the Cardiac Disorders in National Institutes of Health Common Terminology Criteria for Adverse Events NCI CTCAE 4.0 criteria and causality to fluoropyrimidines was assessed according to World Health Organization Uppsala Monitoring Centre (WHO-UMC) guidelines (Supplementary Material S1, available at https://doi.org/10.1016/j.esmoop.2022.100427). Based on clinical records (that included evaluations at the local cardiology unit in most patients), two experienced oncologists [Supplementary Table S2, available at https://doi.org/10.1016/j.esmoop.2022.100427; guided by a cardiologist (AT) as needed] graded cardiac disorders and determined causality, with consensus reached for all patients.

### Patients

All identified patients with solid tumours experiencing grade 1-4 cardiotoxicity during fluoropyrimidine treatment, who were switched to S-1-based therapy, were included. The intended duration of S-1 treatment was until completion of curative treatment or progression on palliative treatment. Of note, complete population-based data on S-1 treatment was available for Tampere and Helsinki University Hospitals, Finland, and for CRCs in Turku, Finland, and cases were included if switch due to cardiotoxicity was the indication. Further, patients with switch due to cardiotoxicity in the RAXO study (NCT01531621) were included. The RAXO study included 1086 Finnish mCRC patients 2012-2018, who were evaluated for metastasectomy and natural course of disease.^19^^,^^20^ Additional cases were retrospectively identified and included from the other participating institutions. The data cut-off for inclusion was 7 October 2020, upon reaching the upper per protocol limit of 200 patients. Data collected included patient characteristics, previous and concurrent cancer therapies with dose intensities, cardiovascular comorbidities, current medications [with anatomical therapeutic chemical (ATC) code], cardiac evaluations, and cardiac treatment during cardiotoxicity. Clinically meaningful non-cardiac adverse events recorded included haematologic toxicity grade 3-4, and non-haematologic toxicity grade 2-4.

### Statistical analyses

The primary endpoint was recurrent cardiotoxicity after switch to S-1-based treatment. The cumulative incidence with its 95% confidence interval (CI) was calculated in a competing risks analysis, where first onset of recurrent cardiotoxicity was the event of interest and stopping of S-1 without recurrent cardiotoxicity a competing risk. It was specified in advance that a probability of recurrent toxicity <15% would be considered clinically meaningful, and 15% thus should not be included in the upper boundary for 95% CI.

An initial estimate for the study was to include 30 local patients. This would have led to an estimated 95% CI of 8.5% to 21.5%, however, which was considered too wide. The study was, therefore, expanded to other centers to include 200 patients, which provided an estimated 95% CI of 12.5% to 17.5%. With a sample size of 200 patients, the power to reject this null hypothesis with a two-sided alpha of 0.05 would be 80% if the true probability was 8% (assuming the power for a test of a proportion approximates that of a cumulative incidence).

Worst grade of cardiotoxicity is presented if multiple events were present. Continuous characteristics are presented as median with range and interquartile range (IQR). Systematic missing information for Dutch patients (*n* = 28) included Eastern Cooperative Oncology Group performance status (ECOG PS), some comorbidities, and survival. Missing values were not imputed. Demographic variables were screened for associations with the crude percentage of recurrent cardiotoxicity with chi-square tests with Bonferroni correction for multiple comparisons, and, if statistically significant differences were noted, odds ratios (OR) and 95% CIs were calculated using logistic regression. Overall survival (OS) from initiation of S-1-based therapy to death of any cause or end of follow-up was estimated using the Kaplan–Meier method.

## Results

### Patients

S-1-based treatment started between 1 November 2011 and 5 October 2020. Data cut-off was 10 May 2021 when median follow-up was 33 months from S-1 initiation, and minimum 50 days. Two hundred patients were included [median age 66 years (range 19-86) and 118 (59%) male]. During the study period, 1118 patients received fluoropyrimidines at Tampere University Hospital, Finland, of whom 74 (7%) experienced cardiotoxicity and 42 (4%) were switched to S-1. At the university hospitals in Turku and Helsinki, Finland, 676 and 1038 patients, respectively, received fluoropyrimidines for mCRC and 27 (4%) and 34 (3%), respectively, were switched to S-1; cardiotoxicity rates, and number of fluoropyrimidine-treated and switch rates are not known for the other centres.

Patient characteristics are presented in Table 1. Before initiation of the cycle of capecitabine/5-FU resulting in cardiotoxicity, 101 (51%) patients had no cardiovascular comorbidities (Table 1; Supplementary Table S3, available at https://doi.org/10.1016/j.esmoop.2022.100427).

**Table 1:** Patient characteristics for all patients at the time of cardiotoxicity with capecitabine or 5-fluorouracil and according to none or recurrence of cardiotoxicity after switch to S-1

	Total		No recurrent cardiotoxicity		Recurrent cardiotoxicity	
	*N* = 200	100%	*n* = 192		*n* = 8	
Age (yrs)						
Median (range)	66	(19-86)	66	(19-86)	64	(51-72)
<70	123	62%	117	61%	6	75%
≥70	77	39%	75	39%	2	25%
Sex						
Female	82	41%	79	41%	3	38%
Male	118	59%	113	59%	5	63%
ECOG						
PS 0	51	26%	47	25%	4	50%
PS 1	105	53%	101	53%	4	50%
PS 2	16	8%	16	8%	0	0%
NA	28	14%	28	15%	0	0%
Cardiovascular comorbidity^a^						
No	101	51%	97	51%	4	50%
Yes	99	50%	95	50%	4	50%
Primary tumour						
Anal cancer	2	1%	2	1%	0	0%
Biliary cancer	3	2%	2	1%	1	13%
Breast cancer	3	2%	3	2%	0	0%
Cancer of unknown primary	2	1%	2	1%	0	0%
Colon cancer	103	52%	99	52%	4	50%
Colon cancer MINEN	1	1%	1	1%	0	0%
Oesophageal cancer	3	2%	3	2%	0	0%
Gastric cancer	20	10%	20	10%	0	0%
Pancreas cancer	5	3%	5	3%	0	0%
Pancreas neuroendocrine	2	1%	2	1%	0	0%
Rectal cancer	55	28%	52	27%	3	38%
Small bowel cancer	1	1%	1	1%	0	0%
Localized or metastatic disease						
Stage I-III	114	57%	110	57%	4	50%
Stage IV	86	43%	82	43%	4	50%
Resection						
Primary tumour	126	64%	119	63%	7	88%
Metastases	14	7%	14	8%	0	0%
Radiotherapy						
Chest wall or breast	4	2%	4	2%	0	0%
Abdomen or pelvis	19	10%	18	9%	1	13%

ECOG PS, Eastern Cooperative Oncology Group performance status; MINEN, mixed neuroendocrine non-neuroendocrine neoplasm.

a Comorbidities and regular medications are specified in Supplementary Table S3, available at https://doi.org/10.1016/j.esmoop.2022.100427.

### Initial cardiotoxicity on capecitabine- or 5-FU-based treatment

Initial cardiotoxicity was associated with capecitabine in 170 (85%) patients, continuous/de Gramont 5-FU in 22 (12%), and bolus 5-FU in 8 (4%) patients. Initial therapy was single-agent fluoropyrimidine in 62 (31%) and combination chemotherapy in the remaining patients (Supplementary Table S4, available at https://doi.org/10.1016/j.esmoop.2022.100427). Biologic therapy was administered to 17% of patients. Chemoradiation for anal or rectal tumours was administered to 13 (7%) patients. Treatment intent was curative in 145 (73%) patients and palliative in 55 (28%). Median relative dose intensity of capecitabine/5-FU was 84% (IQR 69-100) of standard dose and 99% (IQR 81-100) of dose adjusted for age and renal function (Supplementary Table S4, available at https://doi.org/10.1016/j.esmoop.2022.100427).

A single initial cardiac event was diagnosed in 176 (88%) patients, and 24 (12%) patients experienced two to three simultaneous events. The most common cardiotoxicity events (228 events/200 patients) were chest pain considered by the treating oncologist/cardiologist to be a coronary artery spasm-related ‘cardiac event’ in 125 patients (63%), acute coronary syndrome including myocardial infarction in 69 (35%), atrial fibrillation in 8 (4%), other arrhythmias in 12 (6%), heart failure/cardiomyopathy in 7 (4%), and cardiac arrest in 4 (2%) (Table 2).

**Table 2:** Cardiotoxicity during capecitabine or 5-fluorouracil or during switch to S-1-based therapy

	Fluoropyrimidine causing cardiotoxicity						Switch to S-1					
	Total		No recurrent cardiotoxicity		Recurrent cardiotoxicity		Total		No recurrent cardiotoxicity		Recurrent cardiotoxicity	
	*n* = 200	100%	*n* = 192	96%	*n* = 8	4%	*n* = 200	100%	*n* = 192	96%	*n* = 8	4%
Number of cycles to cardiotoxicity or total												
1	153	77%	147	77%	6	75%	18	9%	15	8%	3	38%
2	24	12%	23	12%	1	13%	16	8%	15	8%	1	13%
3	8	4%	8	4%	0	0%	24	12%	23	12%	1	13%
4+^a^	15	8%	14	7%	1	13%	142	71%	139	72%	3	38%
Time to cardiotoxicity onset - regimen												
Median (range) days	5	(0-466)	4.5	(0-209)	5.5	(1-466)	—	—	—	—	16	(6-195)
Time to cardiotoxicity onset - cycle												
Median (range) days	3	(0-41)	3	(0-41)	4.5	(1-11)	—	—	—	—	7	(0-12)
Duration of therapy												
Median (range) days	5	(0-466)	4.5	(0-209)	5.5	(1-466)	147	(6-966)	147	(21-966)	147	(6-357)
Number of cardiotoxicity events												
1	176	88%	170	89%	6	75%	—	—	—	—	8	100%
2	21	11%	20	10%	1	13%	—	—	—	—	0	0%
3	3	2%	2	1%	1	13%	—	—	—	—	0	0%
Cardiotoxicity^b^												
Chest pain^c^	125	63%	122	64%	3	38%	5	3%	—	—	5	63%
Coronary artery syndrome/MI^d^	69	35%	65	34%	4	50%	—	—	—	—	—	—
Atrial fibrillation	8	4%	8	4%	0	0%	—	—	—	—	—	—
Cardiac arrest	4	2%	3	2%	1	13%	—	—	—	—	—	—
Heart failure/cardiomyopathy	7	4%	7	4%	0	0%	—	—	—	—	—	—
Tachycardias	6	3%	3	2%	3	38%	3	2%	—	—	3	38%
Arrhythmia^e^	4	2%	4	2%	0	0%	—	—	—	—	—	—
Bradycardias	2	1%	2	1%	0	0%	—	—	—	—	—	—
Prolonged QT	2	1%	2	1%	0	0%	—	—	—	—	—	—
Hypertension	1	1%	1	1%	0	0%	—	—	—	—	—	—
Worst cardiotoxicity grade												
1	17	9%	16	8%	1	13%	—	—	—	—	6	75%
2	71	36%	68	35%	3	38%	—	—	—	—	2	25%
3	91	46%	88	46%	3	38%	—	—	—	—	0	0%
4	21	11%	20	10%	1	13%	—	—	—	—	0	0%
Action with chemotherapy												
None	—	—	—	—	—	—	2	1%	—	—	2	25%
Dose delayed	—	—	—	—	—	—	1	1%	—	—	1	13%
Temporarily discontinued	8	4%	8	4%	0	0%	2	1%	—	—	2	25%
Permanently discontinued	192	96%	184	96%	8	100%	3	2%	—	—	3	38%
Recovery from cardiac event												
With sequelae	5	3%	4	2%	1	13%	—	—	—	—	0	0%
Without sequelae	195	98%	188	98%	7	88%	—	—	—	—	8	100%
Time to recovery												
Median (range) days	2	(0-274)	2	(0-274)	2	(0-8)	—	—	—	—	1	(0-6)
Time from cardiotoxicity to switch												
Median (range) days	—	—	—	—	—	—	23	(1-3984)	22	(1-3984)	36	(15-870)
Causality^f^												
Not related	0	0%	0	0%	0	0%	—	—	—	—	3	38%
Possibly related	33	17%	32	17%	1	13%	—	—	—	—	3	38%
Probably related	117	59%	112	58%	5	63%	—	—	—	—	2	25%
Related	50	25%	48	25%	2	25%	—	—	—	—	—	—

a 4-14 for cardiotoxicity and 4-46 in total.

b Number of episodes of cardiotoxicity graded according to NCI CTCAE v4.0.

c Chest pain is defined as a disorder characterised by substernal discomfort due to insufficient myocardial oxygenation in National Institutes of Health Common Terminology Criteria for Adverse Events (NCI CTCAE) v4.0.

d ACS/MI, acute coronary syndrome/myocardial infarction.

e Arrythmia was not further specified.

f Causality according to WHO-UMC criteria defined in protocol (Supplementary Material S1, available at https://doi.org/10.1016/j.esmoop.2022.100427).

Diagnostic workup for cardiotoxicity, with one or more modalities, was carried out in all patients, mostly by a cardiologist. There were abnormal clinical findings, such as murmur, irregular pulse, and peripheral oedema, in 10% (18/184 patients having clinical examination at the time of cardiotoxicity), in 64% (116/180) who underwent an electrocardiogram, in 41% (30/74) who underwent an echocardiogram, in 43% (60/141) who had troponin levels measured, in 35% (20/57) who underwent angiography, and in 40% (16/45) who underwent other examinations such as stress test or Holter (details in Supplementary Table S5, available at https://doi.org/10.1016/j.esmoop.2022.100427).

**Figure 1:**
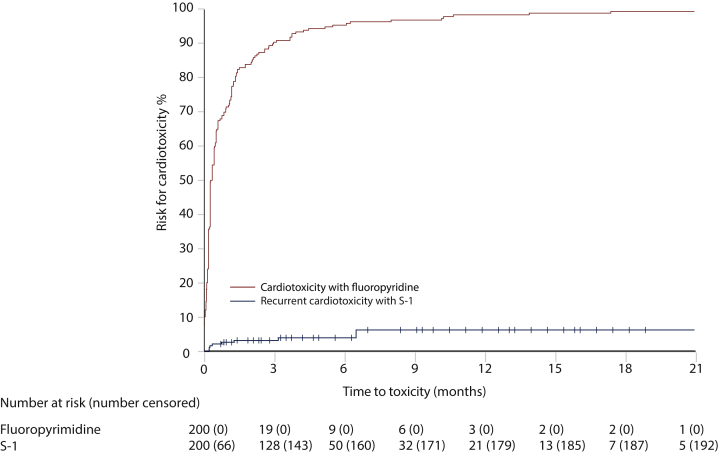
**Time to appearance of cardiotoxicity from initiation of treatment during initial capecitabine or 5-fluorouracil (5-FU)-based therapy (*n* = 200; red) and during S-1-based therapy after switching due to cardiotoxicity on 5-FU or capecitabine (blue)**.

**Figure 2:**
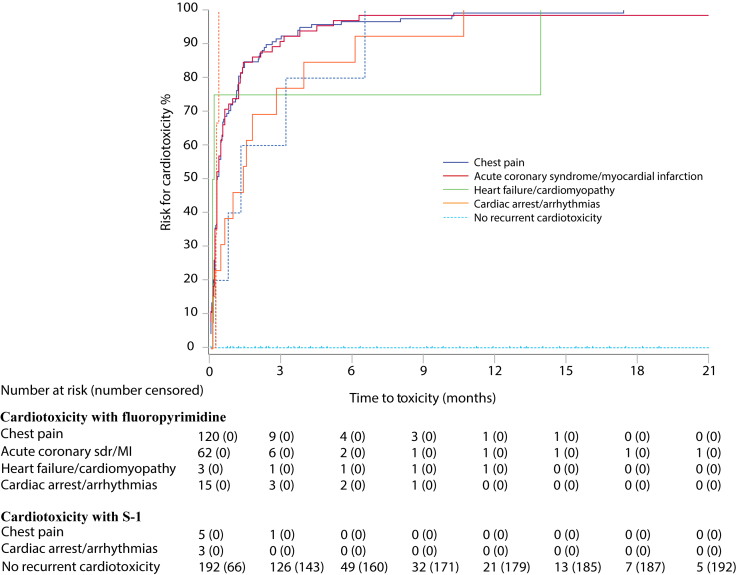
**Time to appearance of worst cardiotoxicity (*n* = 200), i.e. chest pain (blue), acute coronary syndrome/myocardial infarction (red), heart failure/cardiomyopathy (green), or cardiac arrest/arrhythmia (orange) during fluoropyrimidine-based (solid lines) and time to appearance of recurrent cardiotoxicity (*n* = 8) colour-separated as above (dotted lines) and time without recurrent cardiotoxicity (*n* = 192; turquoise) on S-1-based therapy**. MI, myocardial infarction; sdr, syndrome.

The worst grade of cardiotoxicity was severe or life-threatening in 112 (56%) (Table 2). No difference in severity was noted for patients with ongoing medication with ATC codes C01 (cardiac therapy including nitrates) or C08 (calcium channel blockers), that were administered to 28 patients at baseline. Time to onset of cardiotoxicity was median 5 days (IQR 2-16) from initiation of a fluoropyrimidine-containing regimen and appeared during cycle 1 or 2 in 177 (89%) patients (Table 2, Figure 1), without any difference between fluoropyrimidines (data not shown). There were slight differences in time to onset between the cardiac disorders (Figure 2). Cardiac arrest appeared at a median of 2 days (95% CI not evaluable) from treatment initiation, chest pain, both with or without ischemia, on day 4 (95% CI 3-5 days), heart failure/cardiomyopathy on day 5 (0-31 days), atrial fibrillation on day 18 (95% CI 4-32 days), and other arrhythmias on day 41 (95% CI 0-118 days).

As part of their treatment history, rechallenge or change to 5-FU or capecitabine, with prophylactic medication (C08 calcium-channel blocker and/or C01, i.e. nitrates) and/or dose-reduction, was attempted 26 times in 25 patients (13%). Rechallenge with capecitabine was successful in none (0/6; 0%). Change from capecitabine to infused 5-FU was successful in 4/15 (27%), and to bolus 5-FU in 4/5 (80%). Capecitabine/5-FU was permanently discontinued by the treating physician at first cardiotoxic event in 192 (96%) patients (Table 2). The causal relationship according to WHO-UMC between cardiac disorder and fluoropyrimidine treatment was categorized as related/certain in 50 (25%), probable in 117 (59%), and possible in 33 (17%) (Table 2). Median time to recovery from a cardiac event was 2 (IQR 0-4) days, with full recovery in 98%.

Non-cardiac adverse events during initial fluoropyrimidine- and subsequent S-1-based treatments are presented in Supplementary Table S6, available at https://doi.org/10.1016/j.esmoop.2022.100427. Non-haematologic toxicity grade 2-4 was observed in 15% and haematologic toxicity grade 3-4 in 1% during initial fluoropyrimidine treatment, with a short median treatment duration of 5 days (IQR 2-16). DPD status was unknown in 162 patients and was not considered relevant to be tested in 38 patients with grade 2-4 non-cardiac adverse events.

### Switch to S-1-based therapy

Of the 200 Caucasian patients who switched to S-1 therapy, 58 (29%) received S-1 as single-agent therapy (mostly 30 mg/m^2^ twice daily, two weeks out of three), all of whom initially had single-agent capecitabine/5-FU. S-1 (mostly 25 mg/m^2^ twice daily as combination chemotherapy) was administered with the same cytotoxic drug as used initially in 121 (61%) patients and/or with a biologic in 43 (22%), i.e. 3 more patients had combination chemotherapy and 9 more patients had a biologic agent compared with the original capecitabine/5-FU regimen (Supplementary Table S4, available at https://doi.org/10.1016/j.esmoop.2022.100427). In 14 (7%) patients, S-1 was administered as a chemosensitizer during radiotherapy for rectal or anal cancer. Treatment intent after the switch was curative, i.e. adjuvant or neoadjuvant/conversion, in 126 (63%) patients, and the intent was modified from neoadjuvant/conversion to palliative in 18 patients. Median relative dose intensity of S-1 was 92% (IQR 82-100) of standard dose and 100% (IQR 83-100) of dose adjusted for age and renal function (Supplementary Table S4, available at https://doi.org/10.1016/j.esmoop.2022.100427). The median time from the occurrence of cardiotoxicity during capecitabine/5-FU to switch to S-1 was 23 days (IQR 15-98) (Table 2).

One hundred and ninety-two (96%) patients who switched to S-1-based treatment did not experience any recurrence of cardiotoxicity. Cardiotoxicity was observed in eight (4%) patients with 95% CI 2.03-7.89, not including the prespecified upper boundary of 15% and thus, the primary endpoint was met. Cardiotoxicity included chest pain in five and tachycardia in three patients (Table 2) and occurred after a median of 16 days (IQR 7-67) from initiation of S-1 (Table 2, Figure 1). The tachycardia episodes were observed earlier than chest pain (Figure 2). Of the eight patients who experienced recurrent cardiotoxicity, three were on S-1 monotherapy, five on combination therapy with oxaliplatin, and one of these also received bevacizumab (Supplementary Table S4, available at https://doi.org/10.1016/j.esmoop.2022.100427).

Three recurrent cardiotoxicity patients (4%) were observed in the subgroup of 76 patients with the most severe cardiotoxicity on capecitabine/5-FU (grade 3-4 cardiotoxicity, probably related or related/certain causality, cardiotoxicity within 60 days, dose intensity of ≥80%, and permanent discontinuation of capecitabine/5-FU).

Regular C01 (cardiac therapy including nitrates) or C08 (calcium channel blocker) treatment had already been administered to 28 patients before first cardiotoxicity event and continued after the fluoropyrimidine switch. These drugs were administered to an additional 47 patients during the cardiac event on the initial fluoropyrimidine, but their use after switch was not reliably recorded. Of this group of 75 patients known to have used either of these drugs and switched to S-1, 5 patients had recurrent cardiotoxicity whereas 70 did not.

The patients with recurrent cardiotoxicity had a median age of 64 years (range 51-72 years), five were male, and five had cardiovascular comorbidities at baseline (Table 1, Supplementary Table S3, available at https://doi.org/10.1016/j.esmoop.2022.100427). There were no differences in terms of age, gender, ECOG PS, baseline comorbidities predisposing to fluoropyrimidine toxicity, type of S-1-based therapy (including biologics), relative or adjusted dose intensity, or severity of previous cardiotoxicity on the initial fluoropyrimidine between the eight patients with recurrent cardiotoxicity compared with those without; only ischemic heart disease (OR 6.18; 95% CI 1.36-28.11) was more common in patients with recurrent cardiotoxicity (Tables 1 and 2; Supplementary Table S3, available at https://doi.org/10.1016/j.esmoop.2022.100427).

Recurrent cardiotoxicity was grade 1 in six patients and grade 2 in two patients. Details on baseline characteristics, treatments, evaluations, and findings in the eight patients who had recurrent cardiotoxicity are presented in Table 2 and Supplementary Table S7, available at https://doi.org/10.1016/j.esmoop.2022.100427. Five patients experienced chest pain (four of these had previous cardiac comorbidities), and three had tachycardia, one of which was ‘cured’ with appropriate treatment of panic attacks. All recovered without sequelae within a median of 1 (IQR 0-3) day (Table 2). The causal relationship between S-1-based treatment and cardiotoxicity was considered probable in two patients, possible in three, and not related/unlikely in three.

In patients with no recurrent cardiotoxicity (*n* = 192, 96%), median duration of S-1-based treatment was 147 days, for both localised and metastatic disease, during which 139 (72%) received four or more cycles (Table 2, Figure 2). Median duration of S-1-based therapy was also 147 days in the patients with recurrent cardiotoxicity. S-1 was permanently discontinued due to cardiotoxicity in three patients, all receiving adjuvant therapy, and five patients continued treatment with dose reduction in one, temporary discontinuation in two, and no action in two, for 147, 147+, 217+, 336+, and 357 days, respectively.

The successful completion rate with S-1-based treatment was 99% (197 patients). Treatment was discontinued due to completed adjuvant therapy in 88 patients, completed neoadjuvant therapy in 35 (including 14 with chemoradiation), or progressive disease in 74 with palliative therapy, and due to cardiotoxicity in only the 3 patients described in the preceding text. Forty-one patients continued S-1-based treatment after progression by intensification of the regimen, mostly by adding irinotecan (Table 2).

Non-cardiac adverse events during S-1 treatment included grade 3-4 haematologic toxicity in 6% (Supplementary Table S6, available at https://doi.org/10.1016/j.esmoop.2022.100427). Grade 2-4 non-haematologic adverse events occurred in 22%, including neuropathy, nausea, diarrhoea, infection, and hand-foot syndrome.

Median OS from S-1 initiation (*n* = 170) of the patients with localised disease was not reached and was 22 months (95% CI 16-28 months) in patients with metastatic disease, with 5-year survival rates of 78% and 10%, respectively (Supplementary Figure S1, available at https://doi.org/10.1016/j.esmoop.2022.100427). In the largest subgroup with colorectal primary (*n* = 133), median OS was not reached in localised disease and was 26 months (95% CI 22-31 months) in metastatic disease with 5-year survival rates of 83% and 12%, respectively (Supplementary Figure S1, available at https://doi.org/10.1016/j.esmoop.2022.100427).

## Discussion

This retrospective observational cohort study evaluated the feasibility of S-1-based treatment in patients with solid tumours who had experienced cardiotoxicity on capecitabine and infused or bolus 5-FU. The main findings are that almost all patients (99%) who switched to S-1 were able to continue their planned treatment, with 96% of patients not experiencing any recurrence of cardiotoxicity. The main endpoint was met as the 95% CI excluded the prespecified upper boundary of 15% (recurrent cardiotoxicity was 4% with 95% CI 2.02% to 7.89%). Any recurrent cardiotoxicity was of low grade (grades 1-2), allowing continuation of the S-1-based treatment in all but three patients. Indeed, treatment was safely continued using S-1 to the end of scheduled adjuvant treatment in 97%, and in 100% with planned neoadjuvant/conversion intent including chemoradiation and metastasectomy, or until disease progression, with median time on treatment of almost 5 months.

Cardiotoxicity during fluoropyrimidine treatment is a clinical challenge, particularly in patients with gastrointestinal cancers, where it is the cornerstone both in curative and palliative treatments. The evidence-based treatment regimens used in this cohort switched capecitabine or 5-FU to S-1 with companion chemotherapy, biologics, and/or radiotherapy as used initially (Supplementary Tables S1 and S4, available at https://doi.org/10.1016/j.esmoop.2022.100427). Our 5-year survival rates of 78% in localized disease in all cancers, and 83% in CRCs, are in alignment with published adjuvant therapy series and emphasize the high unmet need if capecitabine or 5-FU cannot be used.^21^^,^^22^ Median OS of 22 months in all metastatic cancers and 26 months in mCRC indicate that the efficacy of S-1-based treatment is in line with previous studies in Western and Asian populations (Supplementary Table S1, available at https://doi.org/10.1016/j.esmoop.2022.100427).^21^ Thus, although claims of similar efficacy would require randomized comparisons also in Western populations, and better knowledge of patient and tumour characteristics in our study, there are no indications that S-1-based treatments result in inferior outcomes.

The median dose intensity was 84%-99% (of standard full, or age-/renal function-adjusted dose^23^) for capecitabine- or 5-FU-based treatment, and 92%-100% for S-1-based treatment, thus, dose reductions cannot explain less or milder cardiotoxicity during S-1 treatment. Altogether, the toxicity profile during S-1-based treatment was favourable and in line with previous observations (Supplementary Table S1, available at https://doi.org/10.1016/j.esmoop.2022.100427).^17^ The higher rates of adverse events observed using S-1 compared with capecitabine/5-FU can be explained by the much longer duration of S-1 treatment (median 147 versus 5 days) in this study.

Cardiac comorbidity was present in less than half of the patients who experienced cardiotoxicity. We could not identify patient- or disease-related factors that predicted recurrence of cardiotoxicity, except previous history of ischemic heart disease, which is in line with other studies that explored risk markers for fluoropyrimidine-related cardiotoxicity.^3^^,^^10^^,^^12^^,^^13^^,^^24^

Patients in this study had 5-FU and capecitabine administered with different techniques and with various cytotoxic and biologic drugs. We did not observe any differences in cardiotoxicity with the various combined cytotoxics or biologics. We were not able to identify any differences between the fluoropyrimidines in symptomatology, timing of cardiotoxicity, previous cardiotoxic drugs or radiotherapy, findings in extensive diagnostic work-up, prophylaxis, baseline cardiotoxicity, or other diseases or treatments. Patients were included before the European Medicines Agency (EMA) recommendation for prophylactic DPD testing for fluoropyrimidines (EMA/229267/2020), and none had any clinical indication for on-demand DPD testing. Therefore, we could not investigate the involvement of DPD deficiency and associations with FBAL and other metabolites, the latter with anecdotal correlations to cardiotoxicity and relevance for S-1 mode of action,^12^ as no patients had DPD, FBAL, or other metabolite levels tested.

This study was not designed to provide information on the overall rate of cardiotoxicity in patients receiving fluoropyrimidines, but the proportion of patients switched to S-1 (3%-4% at the three largest centres and a cardiotoxicity rate of 7% at one of the centres) are in line with previous studies that estimated rates of approximately 4%-6% in population-based single-centre studies for breast cancer or CRC patients, or in the Dutch randomized CAIRO studies.^6^, ^7^, ^8^

Several patient series have examined rechallenge, alternative chemotherapy dosing, and regimens to overcome the cardiotoxicity of fluoropyrimidines. Rechallenge, even with concomitant calcium blocker or nitrate prophylaxis, is successful in only 10%-56% of patients,^4^^,^^6^^,^^9^, ^10^, ^11^ and our poor success rate with capecitabine rechallenge is in line with these findings. Baseline regular use of nitrates and calcium-channel blockers (in 14%) was not protective against cardiotoxicity with capecitabine/5-FU, but the prophylactic use of these drugs (in up to 38%) might have reduced recurrent cardiotoxicity during S-1-based therapy. Rechallenge, including prophylaxis, with a 50%-70% reduced dose is successful in 20%-60% of patients with grade 1-2 cardiotoxicities on 5-FU or capecitabine, but the authors advise careful cardiologic monitoring and risk-benefit assessment in light of small patient numbers.^25^ This caution is supported by a study reporting that 5-FU-based regimens can cause silent myocardial ischemia, even in asymptomatic patients.^26^ Rechallenge with capecitabine or 5-FU would thus not be recommended in 56% of our patients with grade 3-4 cardiotoxicity. The success rate of S-1 switch was 96% for this subgroup with severe or life-threatening cardiotoxicity and the strongest causality connections to capecitabine or 5-FU.

Alternatives to S-1 in the switch setting are rare. In two case studies of six and 10 patients, respectively, no subsequent cardiotoxicity was observed after a change to bolus 5-FU-based after cardiotoxicity on infused 5-FU or capecitabine,^27^^,^^28^ which is in line with data from our study. Infused 5-FU is the best companion to cetuximab or panitumumab and the second most common change attempted in our series, but successful in only 27%. Raltitrexed has shown promising results in a small series of mCRC patients experiencing cardiotoxicity due to 5-FU or capecitabine,^29^, ^30^, ^31^, ^32^ but its use has been limited by other toxicities, including mortalities.^29^^,^^31^

Our study has several strengths. The inclusion of 200 patients is by far the largest study presented on this topic. It was an essential part of an S-1 label extension accepted by the EMA: ‘Teysuno, as monotherapy or in combination with oxaliplatin or irinotecan, with or without bevacizumab, is indicated for the treatment of patients with mCRC for whom it is not possible to continue treatment with another fluoropyrimidine due to hand-foot syndrome or cardiovascular toxicity that developed in the adjuvant or metastatic setting’. The comprehensive dataset was systematically collected by experienced investigators in phase I-IV studies that reached consensus decisions on cardiotoxicity grading and causality. The data are also population-based with all S-1 switched patients included from the three largest centres (*n* = 130/200).

Several weaknesses are, however, present. Difficulty in identification of cases might have skewed the findings at smaller centres. In addition, this study has limitations related to its retrospective design, including some missing data for Dutch patients, the fact that causality was not always assessed by the treating physician leading us to cautious judgements, and inclusion of all patients with S-1 switch due to cardiotoxicity regardless of severity and causality. Based on the design of the study, we cannot calculate the rate of cardiotoxicity at the smaller centres, but rates at the three largest centres are in line with reported extensive study- or population-based cardiotoxicity rates.^6^^,^^8^ The survival estimates after S-1 switch are also of unclear relevance given the heterogeneous population, but at least indicate that tumour outcomes were not worse than had the patient not had any cardiotoxicity and continued their initial treatment. A prospective study would have been ideal and likely to provide more complete data. We believe, however, that by collecting a considerable patient population in a relatively short time and using a stringent design, new knowledge of clinical relevance was achieved by this retrospective design.

In conclusion, our study shows that a switch to S-1-based therapy is safe and feasible after development of cardiotoxicity on 5-FU- or capecitabine-based therapy, allowing patients to continue their recommended fluoropyrimidine-based treatment.
