# Supplementary material for: Continuation of fluoropyrimidine treatment with S-1 after cardiotoxicity on capecitabine- or 5-fluorouracil-based therapy in patients with solid tumours: a multicentre retrospective observational cohort study
Source: ESMO Open. 2022 Mar 30;7(3):100427. doi: 10.1016/j.esmoop.2022.100427 (PMC9291631; doi:10.1016/j.esmoop.2022.100427)
Supplement: Supplementary Material [file mmc1.pdf]

Contents

Supplementary Table S1. S-1 and combination regimens in Asian and Caucasian populations. 2
Supplementary Table S2. Participating centres and investigators. 8
Supplementary Table S3. Comorbidities and medications at initiation of cycle with capecitabine or 5-fluorouracil causing cardiac event. 9
Supplementary Table S4. Treatment regimens before and after cardiotoxicity 10
Supplementary Table S5. Cardiac evaluation and treatment for cardiotoxicity during fluoropyrimidine therapy. 11
Supplementary Table S6. Adverse events during treatment with the fluoropyrimidine causing cardiotoxicity and during S-1 based therapy. 12
Supplementary Table S7. Patient characteristics of the eight cases with recurrent cardiotoxicity. 13
Supplementary Figure S1. Overall survival (OS) from S-1 initiation for patients with localized or metastatic solid cancer (panel A), and colorectal cancer (panel B). 14
Supplementary Material S1. Study protocol: Cardioswitch 15

Supplementary Table S1. S-1 and combination regimens in Asian and Caucasian populations.

Regimen	Number of patients	Cancer and setting	Evidence for use in the study	Dosing of S-1 (treatment days/cycle days) Caucasian/Asian	Other drugs	OS months	PFS months	DFS months	Gr 1-2/3-4 toxicity	References
S-1	39	CRC adjuvant	1st line adjuvant CRC	S-1 80-120 mg/day D1-28/42 (4 courses)		3-year 93.6%	ND	3-year 75.5%	Any grade top 4: anorexia 32%, fatigue 27.5%, hyperpigmentation 26.6%, diarrhoea 23.4%; ≥ grade 3: anorexia 4.9%, diarrhoea 4.4%, fatigue 2.4%, nausea 1.6%	Yoshida et al., 2014.[1] Mochizuki et al.,2012.[2]
	13	CRC metastatic	1st line metastatic CRC	S-1 60 mg/m^2 D1-14/21		1/1.5 yr OS 62/41%, not segregated for S-1 alone vs S-1+beva	6.4	ND	Any grade top 4: fatigue 79%, diarrhea 69%, nausea 44%, hypertension 43%. Gr 3 top 4: hypertension 23%, diarrhea 15%, anorexia 13%, fatigue 10%. Gr 4: diarrhea 1%	Kwakman et al., 2017.[3] Kwakman et al., 2019.[4]
	3	Gastric metastatic	1st line adjuvant gastric cancer	S-1 80-120 mg/day D1-21/35		11 months	4 months	ND	Any grade top 4: neutropenia (42%), pigmentation (40%), leucopenia (38%), Anorexia (37%). ≥Grade 3: Neutropenia (11%), anorexia (6%), anaemia (4%), diarrhea (3%)	Koizumi et al., 2008.[5]
	4	Gastric adjuvant/perioperative¥	1st line adjuvant gastric cancer	S-1 80-120 mg/m^2 D1-28/42		80.1% (3yr)	72.2% (RFS)		Grade 3 or 4: anorexia (6.0%), nausea (3.7%), and diarrhea (3.1%)	Sakuramoto et al., 2007.[6]
	2	Breast metastatic	1st line metastatic breast cancer (vs taxane)	S-1 80-120 mg/m^2 D1-28/42		32.7 months	11.2 months		Neutropenia (7%), fatigue (3%) and edema (<1%)	Nishimura et al., 2019.[7]
	1	CUP metastatic	*Treated as gastric metastatic*							
	1	Biliary adjuvant	1st line adjuvant biliary tract cancer	S-1 80-120 mg/day D1-28/42 (4 courses)		NR	NR	NR		Nakachi et al., 2018.[8]
S-1 + bevacizumab	14	CRC metastatic	1st line metastatic CRC	S-1 60 mg/m^2 D1-14/21	Bevacizumab 7.5 mg/kg D1	17 (+/- bevacizumab mixed)	8,4 (+/- bevacizumab mixed)	ND	Any grade top 4: fatigue 79%, diarrhea 69%, nausea 44%, hypertension 43%. Gr 3 top 4: hypertension 23%, diarrhea 15%, anorexia 13%, fatigue 10%. Gr 4: diarrhea 1%	Kwakman et al., 2017.[3] Kwakman et al., 2019.[4]
S-1 + chemoradiation	11	CRC locally advanced rectal cancer	Locally advanced rectal cancer	S-1 80 mg/m^2 D1-14/22-35	RT 50Gy (25 fractions)	ND	ND	ND	Gr 3: 0, Gr 1/2: leukopenia 20.8%, neutropenia 16.7%, anemia 4.2%, thrombocytopenia 8.3%, diarrhea 33.3%, vomiting 20.8%, AST/ALT 4.2%, HFS 0%	Su et al.,2014.[9]
	2	Anal definitive chemoradiation (+mitomycin)	1st line metastatic SCC	S-1 80 mg/m^2 D1-21/rest (2 cycles)	Cisplatin 6 mg/m^2 D1-5/8-12; RT 2 cycles of 30 Gy (2 Gy/day on days 1-5, 8-12, 15-19), 4 wks apart	16 months	ND	0	No grading. Mild perineal skin erosion and appetite loss, no hematologic tox	Nitori et al., 2011.[10]
	1	Gastric adjuvant	1st line adjuvant GC	S-1 80 mg/m^2 D1-5/7 (5 cycles)	RT 45 Gy (25 fractions)	3-year 70%	other determinants	3-year 62.2%	Grade 1/2: leukopenia 71.1%, nausea 63.5%, anorexia 57.7%, fatigue 55.8%; Grade 3/4: leucopenia 11.5%, nausea 9.6%, anorexia 9.6%, vomiting 5.7%	Wang et al., 2018.[11] Park et al., 2021.[12]
S-1 + cisplatin	1	Gastric adjuvant	1st line adjuvant GC	S-1 70 mg/m^2 D1-14/21	Cisplatin 60 mg/m^2 D1	64.4 months	ND	3-year 52.5%	Any grade top 4: nausea 65.4%, anorexia 60.2%, anemia 57.6%, diarrhoea 44.9%; ≥ grade 3: neutropenia 38.5%, anemia 11.5%, anorexia 6.4%, diarrhea/febrile neutropenia 3.8%	Lee et al., 2019.[13]
S-1 + oxaliplatin	42	CRC adjuvant	Adjuvant CRC	S-1 80-120 mg/day D1-14/21 (8 cycles)	Oxaliplatin 130 mg/m^2 D1	3-year 88.0%	3-year 65% (RFS)	3-year 62.7%	Any grade top 5: thrombocytopenia 70.4%, peripheral sensory neuropathy 70.2%, AST 60.4%, leukopenia 58.9%, neutropenia 58.9. Top 4 ≥ Gr 3: neutropenia 17.2%, diarrhea 5.4%, peripheral sensory neuropathy 4.6%, anorexia 3.5%	Kusumoto et al., 2018.[14] Sunami et al., 2020.[15]
	16	CRC metastatic/conversion	1st line mCRC (non-inferority to CapeOX)	S-1 80 mg/m^2 D1-14/21	Oxaliplatin 130 mg/m^2 D1	19	7.1	ND	Grade 3/4: neutropenia 29%, thrombocytopenia 22%, diarrhoea 10%	Hong et al., 2012.[16] Kim et al., 2014.[17]
	5	Pancreas metastatic	2nd line pancreatic metastatic cancer	S-1 80-120 mg/day D1-14/21	Oxaliplatin 100 mg/m^2 D1	7.4 months	3.0 months	ND	All grades: peripheral neuropathy (75.7%), thrombocytopenia 75%, anorexia 69.1%; Grade 3/4: neutropenia (8.1%), thrombocytopenia (10.3%) and anorexia (14.7%)	Ohkawa et al., 2015.[18]
	4	Gastric adjuvant	1st line adjuvant GC	S-1 160 mg/m^2 D1-14/21 (8 cycles)	Oxaliplatin 130 mg/m^2 D1	5-yr OS 78% for neoadj & adj (not differentiated between the two)	ND	ND	Any grade top 5: nausea/vomiting 62%, fatigue 57%, anorexia 57%, weight loss 48%, leukopenia 38%. Grade 3/4: fatigue 24%, nausea/vomiting 5%,	Xue et al., 2018.[19]

									leukopenia 5%, elevated ALT/AST 5%	
	2	Gastric perioperative¥	Neoadjuvant GC	S-1 160 mg/m^2 D1-14/21 (8 cycles)	Oxaliplatin 130 mg/m^2 D1	5-yr OS 78% for neoadj & adj (not differentiated between the two)	ND	ND	Any grade top 5: leukopenia 48%, fatigue 44%, anorexia 40%, nausea/vomiting 40%, diarrhea 40%. Grade 3/4: fatigue 8%, diarrhea 8%, nausea/vomiting 4%, leukopenia 4%	Xue et al., 2018.[19]
	3	Gastric metastatic	1st line advanced GC (SOX vs CS)	S-1 80-120 mg/day D1-14/21	Oxaliplatin 100 mg/m^2 D1	14.1	5.5	ND	Gr >3 top neutropenia 19.5%, anemia 15.1%, hyponatremia 4.4%, sensory neuropathy 4.7%	Yamada et al., 2015.[20] Takahari et al., 2019.[21]
	2	Esophageal metastatic	Adjuvant (following nCRT) EAC	S-1 50 mg/m^2 D1-14/21 (6 cycles)	Oxaliplatin 130 mg/m^2 D1	40.8 (1/2/3 yr OS 85/78/64%)	ND	Recurrence ** FS 28.3	Gr ≥3 top 4: fatigue 88%, peripheral sensory neuropathy 75%, nausea 58%, diarrhea 58%. Gr 1/2 top 4: Nausea 18%, peripheral sensory neuropathy 13%, anorexia 8%, vomiting 8%.	Stroes et al., 2021.[22]
	1	Breast adjuvant	Adjuvant HER-2 neg ER+ breast cancer	S-1 80-120 mg/day D1-14/21	Endocrine therapy: Premenopausal: tamoxifen 20 mg/day or toremifene 40-120 mg/day; Postmenopausal: anastrazole 1 mg/day or letrozole 2.5 mg/day or exemestane 25 mg/day.	ND	ND	89% (52.2 months FU)	Gr >3: decreased neutrophil count (8%), diarrhoea (2%), decreased white blood cells (2%)	Toi et al., 2021.[23]
S-1 + oxaliplatin + epirubicin	2	Gastric perioperative¥	1st line metastatic GC	S-1 80-120mg/day D1-14/21 (3 or 4 cycles)	Oxaliplatin 100 mg/m^2, etoposide 80 mg/m^2, 30 mg/m^2 epirubicin, D1	27.1 months (9.67 w/o resection, 32.95 with resection)	ND	ND	Gr 4 0%. Gr 3: hemoglobin 5.6%, nausea 5.6%, leukocytes 2.8%, vomiting 2.8%, diarrhea 2.8%. Gr 1/2 top 4: nausea 44.4%, vomiting 41.7%, platelets 36.1%, leukocytes 30.6%	Xiang et al., 2020.[24]
S-1 + oxaliplatin + docetaxel	1	Gastric metastatic	1st line metastatic GC	S-1 80 mg/m^2 D1-14/21	Docetaxel 52.5 mg D1, oxaliplatin 105 mg/m^2 D1	12 months	7.6 months	ND	Grade 3/4: neutropenia 37.2%, leukopenia 27.9%, febrile neutropenia 14%, peripheral neuropathy 14%	Kim et al., 2016.[25]
S-1 + oxaliplatin + bevacizumab	20	CRC metastatic/conversion	1st line mCRC (non-inferiority to mFOLFOX6+bevacizumab)	S-1 80-120 mg/m^2 D1-14/21	Oxaliplatin 130 mg/m^2, D1; Beva 7.5 mg/m^2 D1	29.6	12.2	ND	≥ grade 3: neutropenia 9%, diarrhea 9%, anorexia 5%, leucopenia 2%	Baba et al., 2017.[26] Yamada et al., 2013.[27]
	1	CUP metastatic	*Treated as CRC metastatic*							
S-1 + oxaliplatin + trastuzumab	1	Esophageal metastatic	2nd line advanced GC/EGJ	S-1 80-120 mg/day D1-14/21	Oxaliplatin 130 mg/m^2 D1/cycle, trastuzumab 8 mg - 6 mg/kg/cycle	18.1	8.8	ND	Any grade: anemia 96%, peripheral sensory neuropathy 84%, thrombocytopenia/neutropenia 78.7%; grade 3/4: peripheral sensory neuropathy 16%, neutropenia 10.7%, anemia/diarrhea 6.7%	Takahari et al., 2019.[21] Ter Veer et al., 2018.[28]
S-1 + irinotecan	3	CRC metastatic	2nd line metastatic CRC (non-inferiority to FOLFIRI)	S-1 80-120 mg/day D1-14/28	Irinotecan 125 mg/m^2, D1,15,	17.8	5.8	ND	All grades: diarrhea 79.5%, anemia 74.3%, leucopenia 73.3%, fatigue, 72.9%; grade 3/4: neutropenia 36.2%, diarrhea 20.5%, leucopenia 18.1%, anorexia 11%	Muro et al., 2010.[29] Yasui et al., 2015.[30]
S-1 + irinotecan	13	CRC metastatic (elderly)	2nd line metastatic CRC	S-1 40 mg/m^2 D1-14/21	irinotecan 180 mg/m^2 D1	ND	ND	ND	ND	Winther et al, 2019[31]
	1	Pancreas metastatic	2nd line metastatic pancreas	S-1 80-120 mg/day D1-14/28	irinotecan 100 mg/m^2 D1&15	6.8 months	3.5 months	ND	All grades: anorexia (75.0%), nausea 70.3%, diarrhea 67.2%, leucopenia 64.1%; grade ≥3: anorexia 23.4%, neutropenia/anemia 15.6%, leucopenia 14.1%	Ioka et al., 2017.[32]
	1	Biliary metastatic	*Treated as pancreas metastatic*							
S-1 + irinotecan + bevacizumab	6	CRC metastatic	2nd line metastatic CRC	S-1 80 mg/m^2 D1-14/28	Irinotecan 100 or 150 mg/m^2 D1, bevacizumab 5 or 7.5 mg/kg D1	34.9	14	ND	Any grade top 5: leukopenia 65.7%, neutropenia 62.8%, diarrhea 62.3%, anorexia 59.8%, alopecia 59.8%. Gr ≥3 top 4: neutropenia 24.3%, diarrhea 13.4, leukopenia 8.8, hypertension 8.4%	Sadahiro, 2020.[33] Yamada et al., 2018.[34]
S-1 + irinotecan + bevacizumab	11	CRC metastatic (elderly)	2nd line metastatic CRC	S-1 40 mg/m^2 D1-14/21	irinotecan 180 mg/m^2 D1	ND	ND	ND	ND	Winther et al, 2019[31]
	1	Small bowel metastatic	*treated as CRC metastatic*							
S-1 + irinotecan + cetuximab	1	CRC metastatic	1st line metastatic CRC	S-1 80 mg/m^2 D1-14/28	Irinotecan 100 mg/m^2 D1&15 + cetuximab 400 mg/m^2 in week1 and 250 mg/m^2 in subsequent weeks	25.8	11.3		Gr 3/4 top 4: neutropenia 43, leukopenia 20, dry skin 20, acne-like rash 13%	Eto et al., 2015.[35] Samura et al., 2020.[36]
S-1 + temozolomide	3	NET/NEC metastatic/conversion (pancreas/CRC)	2nd line NETs (retrospective cohort)	S-1 80 mg/m^2 D1-14/21	Temozolomide 200 mg D10-14/21	ND	not achieved	(PR+SD 80% (16/20 ptns)	Grade 1/2: leukopenia (n = 6, 30%), neutropenia (n = 4, 20%), nausea (n = 8, 40%), vomiting (n = 3, 15%),); ≥ 3: vomiting 5% (n=1)	Zhao et al., 2018.[37]
S-1 + gemcitabine	1	Pancreas metastatic/neoadjuvant	1st line metastatic pancreatic cancer	S-1 80-120 mg/day D1-14/21	Gemcitabine 1000 mg/m^2 D1+D8/21	10.1 months	5.7	ND	Gr≥ 3 top 4: leucopenia 37.8%, neutropenia 62.2%, thrombocytopenia 17.2%, Hb decrease 17.2%	Ueno et al., 2013.[38]

	1	Biliary metastatic	1st line advanced/ recurrent biliary tract cancer	S-1 60-100 mg/day D1-14/21	Gemcitabine 1000 mg/m^2 D1+D8/21	15.1 month	6.8 months		Any grade: anemia 98.3%, neutropenia 86.4%; leucopenia and thrombocytopenia 77.4%; grade 3/4: neutropenia 59.9%, leucopenia 24.9%, biliary tract infection 20.9%	Morizane et al., 2019.[39]
**total treatment lines**	**244**									

¥Perioperative = neoadjuvant +/- adjuvant
CUP=Cancer of unknown primary, ND = not determined; NR = not reported

Recommended dose equivalences of S-1 in Asian and Caucasian populations in colorectal cancer.

The majority of patients in this study were treated for colorectal cancer. The recommended doses of S-1 were determined by phase 1 studies (dose finding) and their efficacy and safety were confirmed by phase 2 studies and later.

a. Single agent of S-1

[Asian[6,40]]
Recommended dose of S-1 is 40, 50, and 60 mg/body according to the following BSA range.
S-1 is administered twice daily, after breakfast and after the evening meal, for 28 consecutive days, followed by a 14-day rest.

Body surface area (m^2)	Dose (tegafur equivalent)
< 1.25	40 mg
1.25 - < 1.5	50 mg
≥ 1.5	60 mg

[Caucasian[3,41]]
Recommended dose of S-1 is 30 mg/m^2.
S-1 is administered twice daily, at least one hour before or one hour after meal, for 14 consecutive days, followed by a 7-day rest.

b. Combination with oxaliplatin

S-1/oxaliplatin (+ bevacizumab) regimen

[Asian[16,27,42,43]]
Recommended dose of S-1 is 40, 50, and 60 mg/body according to the following BSA range.
S-1 is administered twice daily, after breakfast and after the evening meal, for 28 consecutive days, followed by a 14-day rest.

Body surface area (m^2)	Dose (tegafur equivalent)
< 1.25	40 mg
1.25 - < 1.5	50 mg
≥ 1.5	60 mg

Recommended dose of oxaliplatin is 130 mg/m^2 on day 1, every 3 weeks.
Recommended dose of bevacizumab is 7.5 mg/kg on day 1 in case of adding bevacizumab to S-1/oxaliplatin regimen.

[Caucasian[44]]
Recommended dose of S-1 is 25 mg/m^2.
S-1 is administered twice daily, at least one hour before or one hour after meal, for 14 consecutive days, followed by a 7-day rest.
Recommended dose of oxaliplatin is 130mg/m^2 on day 1, every 3 weeks.
Recommended dose of bevacizumab is 7.5 mg/kg on day 1, every 3 weeks.

c. Combination with irinotecan

S-1/irinotecan regimen

[Asian[29,45]]
Recommended dose of S-1 is 40, 50, and 60 mg/body according to the following BSA range.
S-1 is administered twice daily, after breakfast and after the evening meal, for 14 consecutive days, followed by a 14-day rest.

Body surface area (m^2)	Dose (tegafur equivalent)
< 1.25	40 mg
1.25 - < 1.5	50 mg
≥ 1.5	60 mg

Recommended dose of irinotecan is 125 mg/m^2 on days 1 and 15, every 4 weeks

[Caucasian[46]]
Recommended dose of S-1 is 25 mg/m^2.
S-1 is administered twice daily, at least one hour before or one hour after meal, for 14 consecutive days, followed by a 7-day rest.
Recommended dose of irinitecan is 100mg/m^2 on day 1, every 3 weeks.

S-1/irinotecan + Bevacizumab regimen

[Asian[34, 47–50]]
3-week regimen:
Recommended dose of S-1 is 40, 50 and 60 mg/body according to the following BSA range.
S-1 is administered twice daily, after breakfast and after the evening meal, for 14 consecutive days, followed by a 7-day rest.

Body surface area (m^2)	Dose (tegafur equivalent)
< 1.25	40 mg
1.25 - < 1.5	50 mg
≥ 1.5	60 mg

Recommended dose of irinotecan is 150 mg/m^2 on day 1, every 3 weeks
Recommended dose of bevacizumab is 7.5 mg/kg on day 1, every 3 weeks.
4-week regimen:
Recommended dose of S-1 is 40, 50, and 60 mg/body according to the following BSA range.
S-1 is administered twice daily, after breakfast and after the evening meal, for 14 consecutive days, followed by a 14-day rest.

Body surface area (m^2)	Dose (tegafur equivalent)
< 1.25	40 mg
1.25 - < 1.5	50 mg
≥ 1.5	60 mg

Recommended dose of irinotecan is 100 mg/m^2 on days 1 and 15, every 4 weeks
Recommended dose of bevacizumab is 5.0 mg/kg on days 1 and 15, every 4 weeks.

[Caucasian]
No data is available.

References

1. Yoshida M, Ishiguro M, Ikejiri K et al. S-1 as adjuvant chemotherapy for stage III colon cancer: a randomized phase III study (ACTS-CC trial). Ann Oncol 2014;25:1743-1749.
2. Mochizuki I, Takiuchi H, Ikejiri K et al. Safety of UFT/LV and S-1 as adjuvant therapy for stage III colon cancer in phase III trial: ACTS-CC trial. Br J Cancer 2012;106:1268-1273.
3. Kwakman JJM, Simkens LHJ, van Rooijen JM et al. Randomized phase III trial of S-1 versus capecitabine in the first-line treatment of metastatic colorectal cancer: SALTO study by the Dutch Colorectal Cancer Group. Ann Oncol 2017;28:1288-1293.
4. Kwakman JJM, van Werkhoven E, Simkens LHJ et al. Updated Survival Analysis of the Randomized Phase III Trial of S-1 Versus Capecitabine in the First-Line Treatment of Metastatic Colorectal Cancer by the Dutch Colorectal Cancer Group. Clin Colorectal Cancer 2019;18:e229-e230.
5. Koizumi W, Narahara H, Hara T et al. S-1 plus cisplatin versus S-1 alone for first-line treatment of advanced gastric cancer (SPIRITS trial): a phase III trial. Lancet Oncol 2008;9:215-221.
6. Sakuramoto S, Sasako M, Yamaguchi T et al. Adjuvant chemotherapy for gastric cancer with S-1, an oral fluoropyrimidine. N Engl J Med 2007;357:1810-1820.
7. Nishimura R, Mukai H, Uemura Y et al. Evaluation of oral S-1 as a first-line chemotherapy for metastatic HER2-negative breast cancer: An analysis of two randomized phase III studies (SELECT BC-CONFIRM and SELECT BC). J Clin Oncol 2019; 37:15_suppl, 1083.
8. Nakachi K, Konishi M, Ikeda M et al. A randomized Phase III trial of adjuvant S-1 therapy vs. observation alone in resected biliary tract cancer: Japan Clinical Oncology Group Study (JCOG1202, ASCOT). Jpn J Clin Oncol 2018;48:392-395.
9. Su M, Zhu LC, Wei HP, Luo WH, Lin RF, Zou CL. S-1-Based versus capecitabine-based preoperative chemoradiotherapy in the treatment of locally advanced rectal cancer: a matched-pair analysis. PLoS One 2014;9:e106162.
10. Nitori N, Kato Y, Kato A et al. Clinical experience with chemoradiotherapy comprising S-1 plus low-dose cisplatin in a patient with stage IV anal cancer. Anticancer Res 2011;31:3983-3989.
11. Wang X, Zhao DB, Yang L et al. S-1 chemotherapy and intensity-modulated radiotherapy after D1/D2 lymph node dissection in patients with node-positive gastric cancer: a phase I/II study. Br J Cancer 2018;118:338-343.
12. Park SH, Lim DH, Sohn TS et al. A randomized phase III trial comparing adjuvant single-agent S1, S-1 with oxaliplatin, and postoperative chemoradiation with S-1 and oxaliplatin in patients with node-positive gastric cancer after D2 resection: the ARTIST 2 trial☆. Ann Oncol 2021;32:368-374.
13. Lee CK, Jung M, Kim HS et al. S-1 Based Doublet as an Adjuvant Chemotherapy for Curatively Resected Stage III Gastric Cancer: Results from the Randomized Phase III POST Trial. Cancer Res Treat 2019;51:1-11.
14. Kusumoto T, Sunami E, Ota M et al. Planned Safety Analysis of the ACTS-CC 02 Trial: A Randomized Phase III Trial of S-1 With Oxaliplatin Versus Tegafur and Uracil With Leucovorin as Adjuvant Chemotherapy for High-Risk Stage III Colon Cancer. Clin Colorectal Cancer 2018;17:e153-e161.
15. Sunami E, Kusumoto T, Ota M et al. S-1 and Oxaliplatin Versus Tegafur-uracil and Leucovorin as Postoperative Adjuvant Chemotherapy in Patients With High-risk Stage III Colon Cancer (ACTS-CC 02): A Randomized, Open-label, Multicenter, Phase III Superiority Trial. Clin Colorectal Cancer 2020;19:22-31.e6.
16. Hong YS, Park YS, Lim HY et al. S-1 plus oxaliplatin versus capecitabine plus oxaliplatin for first-line treatment of patients with metastatic colorectal cancer: a randomised, non-inferiority phase 3 trial. Lancet Oncol 2012;13:1125-1132.
17. Kim, ST, Hong, YS, Lim, HY et al. S-1 plus oxaliplatin versus capecitabine plus oxaliplatin for the first-line treatment of patients with metastatic colorectal cancer: updated results from a phase 3 trial. BMC Cancer 2014;14;883.
18. Ohkawa S, Okusaka T, Isayama H et al. Randomised phase II trial of S-1 plus oxaliplatin vs S-1 in patients with gemcitabine-refractory pancreatic cancer. Br J Cancer 2015;112:1428-1434.
19. Xue K, Ying X, Bu Z et al. Oxaliplatin plus S-1 or capecitabine as neoadjuvant or adjuvant chemotherapy for locally advanced gastric cancer with D2 lymphadenectomy: 5-year follow-up results of a phase II-III randomized trial. Chin J Cancer Res 2018;30:516-525.
20. Yamada Y, Higuchi K, Nishikawa K et al. Phase III study comparing oxaliplatin plus S-1 with cisplatin plus S-1 in chemotherapy-naïve patients with advanced gastric cancer. Ann Oncol 2015;26:141-148.
21. Takahari D, Chin K, Ishizuka N et al. Multicenter phase II study of trastuzumab with S-1 plus oxaliplatin for chemotherapy-naïve, HER2-positive advanced gastric cancer. Gastric Cancer 2019;22:1238-1246.
22. Stroes CI, Schokker S, Molenaar RJ et al. A Phase II Study Demonstrates No Feasibility of Adjuvant Treatment with Six Cycles of S-1 and Oxaliplatin in Resectable Esophageal Adenocarcinoma, with ERCC1 as Biomarker for Response to SOX. Cancers 2021;13:839.
23. Toi M, Imoto S, Ishida T et al. Adjuvant S-1 plus endocrine therapy for oestrogen receptor-positive, HER2-negative, primary breast cancer: a multicentre, open-label, randomised, controlled, phase 3 trial. Lancet Oncol 2021;22:74-84.
24. Xiang XS, Su Y, Li GL, Ma L, Zhou CS, Ma RF. Phase II Study of Preoperative Intra-Arterial Epirubicin, Etoposide, and Oxaliplatin Combined with Oral S-1 Chemotherapy for the Treatment of Borrmann Type 4 Gastric Cancer. J Gastric Cancer 2020;20:395-407.
25. Kim HS, Ryu MH, Zang DY et al. Phase II study of docetaxel, oxaliplatin, and S-1 therapy in patients with metastatic gastric cancer. Gastric Cancer 2016;19:579-585.
26. Baba H, Yamada Y, Takahari D et al. S-1 and oxaliplatin (SOX) plus bevacizumab versus mFOLFOX6 plus bevacizumab as first-line treatment for patients with metastatic colorectal cancer: updated overall survival analyses of the open-label, non-inferiority, randomised phase III: SOFT study. ESMO Open 2017;2:e000135.
27. Yamada Y, Takahari D, Matsumoto H et al. Leucovorin, fluorouracil, and oxaliplatin plus bevacizumab versus S-1 and oxaliplatin plus bevacizumab in patients with metastatic colorectal cancer (SOFT): an open-label, non-inferiority, randomised phase 3 trial. Lancet Oncol 2013;14:1278-1286.
28. Ter Veer E, Creemers A, de Waal L, van Oijen MGH, van Laarhoven HWM. Comparing cytotoxic backbones for first-line trastuzumab-containing regimens in human epidermal growth factor receptor 2-positive advanced oesophagogastric cancer: A meta-analysis. Int J Cancer 2018;143:438-448.
29. Muro K, Boku N, Shimada Y et al. Irinotecan plus S-1 (IRIS) versus fluorouracil and folinic acid plus irinotecan (FOLFIRI) as second-line chemotherapy for metastatic colorectal cancer: a randomised phase 2/3 non-inferiority study (FIRIS study). Lancet Oncol 2010;11:853-860.
30. Yasui H, Muro K, Shimada Y et al. A phase 3 non-inferiority study of 5-FU/l-leucovorin/irinotecan (FOLFIRI) versus irinotecan/S-1 (IRIS) as second-line chemotherapy for metastatic colorectal cancer: updated results of the FIRIS study. J Cancer Res Clin Oncol 2015;141:153-160.
31. Winther SB, Liposits G, Skuladottir H et al. Reduced-dose combination chemotherapy (S-1 plus oxaliplatin) versus full-dose monotherapy (S-1) in older vulnerable patients with metastatic colorectal cancer (NORDIC9): a randomised, open-label phase 2 trial. Lancet Gastroenterol Hepatol 2019;4:376-388.
32. Ioka T, Komatsu Y, Mizuno N et al. Randomised phase II trial of irinotecan plus S-1 in patients with gemcitabine-refractory pancreatic cancer. Br J Cancer 2017;116:464-471.
33. Sadahiro S, Suzuki T, Okada K et al. Oral S-1 with 24-h Infusion of Irinotecan plus Bevacizumab versus FOLFIRI plus Bevacizumab as First-Line Chemotherapy for Metastatic Colorectal Cancer: An Open-Label Randomized Phase II Trial. Oncology 2020;98:637-642.
34. Yamada Y, Denda T, Gamoh M et al. S-1 and irinotecan plus bevacizumab versus mFOLFOX6 or CapeOX plus bevacizumab as first-line treatment in patients with metastatic colorectal cancer (TRICOLORE): a randomized, open-label, phase III, noninferiority trial. Ann Oncol 2018;29:624-631.
35. Eto T, Masuishi T, Matsui T et al. P-278 A phase II study of cetuximab in combination with irinotecan plus S-1 as first-line treatment in patients with KRAS wild-type metastatic colorectal cancer (CIRIS study): overall survival analysis. Ann Oncol 2015;26 (Suppl 4):IV82.
36. Samura H, Oki E, Okumura H et al. A phase I/II study of S-1 and irinotecan (IRIS) combined with cetuximab in patients with RAS wild-type metastatic colorectal cancer (KSCC1401). Cancer Chemother Pharmacol 2020;86:285-294.
37. Zhao J, Zhao H, Chi Y. Safety and Efficacy of the S-1/Temozolomide Regimen in Patients with Metastatic Neuroendocrine Tumors. Neuroendocrinology 2018;106:318-323.
38. Ueno H, Ioka T, Ikeda M et al. Randomized phase III study of gemcitabine plus S-1, S-1 alone, or gemcitabine alone in patients with locally advanced and metastatic pancreatic cancer in Japan and Taiwan: GEST study. J Clin Oncol 2013;31:1640-1648.
39. Morizane C, Okusaka T, Mizusawa J et al. Combination gemcitabine plus S-1 versus gemcitabine plus cisplatin for advanced/recurrent biliary tract cancer: the FUGA-BT (JCOG1113) randomized phase III clinical trial. Ann Oncol 2019;30:1950-1958.
40. Boku N, Yamamoto S, Fukuda H et al. Fluorouracil versus combination of irinotecan plus cisplatin versus S-1 in metastatic gastric cancer: a randomised phase 3 study. Lancet Oncol 2009;10:1063-1069.
41. Zhu AX, Clark JW, Ryan DP et al. Phase I and pharmacokinetic study of S-1 administered for 14 days in a 21-day cycle in patients with advanced upper gastrointestinal cancer. Cancer Chemother Pharmacol 2007;59:285-293.
42. Yamada Y, Tahara M, Miya T et al. Phase I/II study of oxaliplatin with oral S-1 as first-line therapy for patients with metastatic colorectal cancer. Br J Cancer 2008;98:1034-1038.
43. Zang DY, Lee BH, Park HC et al. Phase II study with oxaliplatin and S-1 for patients with metastatic colorectal cancer. Ann Oncol 2009;20:892-896.
44. Chung KY, Saito K, Zergebel C, Hollywood E, Segal M, Saltz LB. Phase I study of two schedules of oral S-1 in combination with fixed doses of oxaliplatin and bevacizumab in patients with advanced solid tumors. Oncology 2011;81:65-72.
45. Komatsu Y, Yuki S, Fuse N et al. Phase 1/2 clinical study of irinotecan and oral S-1 (IRIS) in patients with advanced gastric cancer. Adv Ther 2010;27:483-492.

46. Winther SB, Zubcevic K, Qvortrup C et al. Experience with S-1 in older Caucasian patients with metastatic colorectal cancer (mCRC): Findings from an observational chart review. Acta Oncol 2016;55:881-885.
47. Yamada Y, Yasui H, Goto A et al. Phase I study of irinotecan and S-1 combination therapy in patients with metastatic gastric cancer. Int J Clin Oncol 2003;8:374-380.
48. Goto A, Yamada Y, Yasui H et al. Phase II study of combination therapy with S-1 and irinotecan in patients with advanced colorectal cancer. Ann Oncol 2006;17:968-973.
49. Yamada Y, Yamaguchi T, Matsumoto H et al. Phase II study of oral S-1 with irinotecan and bevacizumab (SIRB) as first-line therapy for patients with metastatic colorectal cancer. Invest New Drugs 2012;30:1690-1696.
50. Komatsu Y, Yuki S, Sogabe S et al. Phase II study of combined chemotherapy with irinotecan and S-1 (IRIS) plus bevacizumab in patients with inoperable recurrent or advanced colorectal cancer. Acta Oncol 2012;51:867-872.

Supplementary Table S2. Participating centres and investigators.

Country	Centre	Patients	Data entry, causality and grading
All patients		200	Pia Osterlund (data verification)
			Sampsa Kinos (data verification)
			Arco Teske (data verification cardiology)
Finland	Helsinki University Hospital	61	Leena-Maija Soveri
			Päivi Halonen
	Tampere University Hospital	42	Tapio Salminen
			Maarit Bärlund
	Turku University Hospital	27	Eetu Heervä
			Annika Ålgars
			Raija Ristamäki
	Oulu University Hospital	3	Raija Kallio (Pia Osterlund)
The Netherlands	Amsterdam University Medical Centre	30	Robert-Jan Kwakmann
			Cornelis Punt
Ireland	St Vincent’s University Hospital	11	Ray McDermott
			Mary O´Reilly
Denmark Herning	Odense University Hospital	7	Per Pfeiffer (Pia Osterlund)
	Regional Hospital West Jutland	3	Gabor Liposits (Pia Osterlund)
Sweden	Skane University Hospital	7	Helga Hagman (Pia Osterlund)
	Uppsala Akademiska	4	Rebecka Röckert
			Bengt Glimelius
	Karolinska University Hospital	3	Jan-Erik Frödin
			Carl-Henrik Shah
	Sundsvall University Hospital	1	Petra Flygare (Pia Osterlund)
Norway Bergen	Bergen University Hospital	1	Halfdan Sorbye (Pia Osterlund)

Supplementary Table S3. Comorbidities and medications at initiation of cycle with capecitabine or 5-fluorouracil causing cardiac event.

		Total		No recurrent cardiotoxicity		Recurrent cardiotoxicity	
		n=200	%	n=192	%	n=8	%
Cardiovascular comorbidity		99	50%	95	50%	4	50%
	Hypertension	79	40%	75	39%	4	50%
	Ischemic heart disease	20	10%	17	9%	3	38%*
	Arrythmia	13	7%	13	7%	0	0%
	Cerebrovascular event	7	4%	7	4%	0	0%
	Myocardial infarction	4	2%	4	2%	0	0%
	Aortic or valve disorders	3	2%	3	2%	0	0%
	Atherosclerosis	3	2%	3	2%	0	0%
	Cardiac heart failure	3	2%	3	2%	0	0%
	Cardiomyopathy	2	1%	1	1%	1	13%
	Arterial or venous thromboembolism	2	1%	2	1%	0	0%
	RBBB or LBBB or prolonged QT	2	1%	2	1%	0	0%
	APC Resistance	2	1%	2	1%	0	0%
Metabolic comorbidity		58	29%	55	29%	3	38%
	Dyslipidaemia	41	21%	40	21%	1	13%
	Diabetes mellitus type 2	18	9%	16	8%	2	25%
	Obesity	9	5%	9	5%	0	0%
Renal comorbidity		7	4%	7	4%	0	0%
	Mild (CC 50-85mL/min)	5	3%	5	3%	0	0%
	Normal (CC>85mL/min)	2	1%	2	1%	0	0%
Lung comorbidity		9	5%	9	5%	0	0%
	COPD	6	3%	6	3%	0	0%
	Asthma	3	2%	3	2%	0	0%
Other comorbidity		70	35%	65	34%	5	63%
	Second malignancy	17	9%	14	7%	3	38%
	Hypothyroidism	14	7%	13	7%	1	13%
	Benign prostate hyperplasia	9	5%	9	5%	0	0%
	Psychiatric disorders	9	5%	8	4%	1	13%
	Gastro-oesophageal reflux disease	8	4%	8	4%	0	0%
	Musculoskeletal disorders	8	4%	8	4%	0	0%
	Gout	5	3%	4	2%	1	13%
	Glaucoma	4	2%	4	2%	0	0%
	Skin disorder	3	2%	3	2%	0	0%
	Inflammatory bowel disease	3	2%	3	2%	0	0%
	Sleep apnoea	2	1%	2	1%	0	0%
	Amyloidosis	1	1%	1	1%	0	0%
	Anaemia	1	1%	1	1%	0	0%
	Autoimmune Sarcoidosis	1	1%	1	1%	0	0%
	Brain injury	1	1%	1	1%	0	0%
	Coeliac disease	1	1%	1	1%	0	0%
	Migraine	1	1%	1	1%	0	0%
	Multiple sclerosis	1	1%	1	1%	0	0%
	Parkinson's disease	1	1%	1	1%	0	0%
	Polyneuropathy	1	1%	1	1%	0	0%
	Sjogren's syndrome	1	1%	1	1%	0	0%
	Vertigo	1	1%	1	1%	0	0%
Cardiovascular medication	ATC C Cardiovascular system	88	44%	84	44%	4	50%
	C01 Cardiac Therapy	7	4%	7	4%	0	0%
	C03 Diuretics	10	5%	9	5%	1	13%
	C05 Vasoprotective	1	1%	1	1%	0	0%
	C07 Beta Blocking Agents	34	17%	34	18%	0	0%
	C08 Calcium Channel Blockers	22	11%	22	12%	0	0%
	C09 Renin Angiotensin System	53	27%	51	27%	2	25%
	C10 Lipid Modifying Agents	42	21%	41	21%	1	13%
Other medication	ATC miscellaneous						
	A02 Drugs for acid related disorders	59	30%	56	29%	3	38%
	A10 Drugs used in diabetes	16	8%	14	7%	2	25%
	B01 Antithrombotic medication	42	21%	40	21%	2	25%
	B03 Antianaemic preparations	11	6%	11	6%	0	0%
	M01 Anti-inflammatory drugs	5	3%	5	3%	0	0%
	N02 Analgesics	34	17%	33	17%	1	13%

ATC, anatomical therapeutic chemical code; * OR 6.18 (95%CI 1.36–28.11)

Supplementary Table S4. Treatment regimens before and after cardiotoxicity

		Fluoropyrimidine causing cardiotoxicity						Switch to S-1					
		Total		No recurrent cardiotoxicity		Recurrent cardiotoxicity		Total		No recurrent cardiotoxicity		Recurrent cardiotoxicity	
		n=200	100%	n=192	96%	n=8	4%	n=200	100%	n=192	96%	n=8	4%
Fluoropyrimidine	Capecitabine	170	85%	163	85%	7	88%						
	Continuous / de Gramont	22	12%	21	12%	1	13%						
	Bolus	8	4%	8	4%	0	0%						
	S-1							200	100%	192	96%	8	4%
Chemotherapy	Single fluoropyrimidine	62	31%	131	32%	1	13%	58	29%	55	29%	3	38%
	Docetaxel	6	3%	6	3%	0	0%	1	1%	1	1%	0	0%
	Epirubicin	5	3%	5	3%	0	0%	2	1%	2	1%	0	0%
	Oxaliplatin	107	54%	100	52%	7	88%	99	50%	94	49%	5	63%
	Cisplatin	2	1%	2	1%	0	0%	1	1%	1	1%	0	0%
	Carboplatin	0	0%	0	0%	0	0%	1	1%	1	1%	0	0%
	Irinotecan	6	3%	6	3%	0	0%	16	8%	16	8%	0	0%
	Gemcitabine	2	1%	2	1%	0	0%	1	1%	1	1%	0	0%
	Mitomycin C	1	1%	1	1%	0	0%	1	1%	1	1%	0	0%
	Temozolamide	2	1%	2	1%	0	0%	3	2%	3	2%	0	0%
Biologic combined	No	166	83%	161	84%	5	63%	157	79%	150	78%	7	88%
	Trastuzumab	1	1%	1	1%	0	0%	1	1%	1	1%	0	0%
	Bevacizumab	29	15%	26	14%	3	38%	40	20%	39	20%	1	13%
	Cetuximab	2	1%	2	1%	0	0%	1	1%	1	1%	0	0%
	Panitumumab	0	0%	0	0%	0	0%	1	1%	1	1%	0	0%
	Octreotide	1	1%	1	1%	0	0%	1	1%	1	1%	0	0%
Radiotherapy combined	Pelvic	13	7%	13	7%	0	0%	14	7%	14	7%	0	0%
Treatment intent	Adjuvant	92	46%	88	46%	4	50%	91	46%	87	45%	4	50%
	Neo-adjuvant	53	27%	51	27%	2	25%	35	18%	33	17%	2	25%
	First line	46	23%	44	23%	2	25%	54	27%	53	28%	1	13%
	Second line	7	4%	7	4%	0	0%	12	6%	11	6%	1	13%
	Third or Later line	2	1%	2	1%	0	0%	8	4%	8	4%	0	0%
Dose Intensity single	Median mg/m2/day (range)	¥1636	(400*-3000¤)	¥1636	(400*-3000¤)	¥2500	(¥2500-¥2500)	50	(33-61)	50	(33-61)	53	(42-60)
	Dose of standard regimen∫	69	(40-101)	69	(40-101)	100	(100-100)	83%	(55-102%)	83%	(55-102%)	88%	(82-100%)
	Adjusted dose of standard regimen₵	86	(40-101)	86	(40-101)	100	(100-100)	98%	(55-106%)	98%	(55-106%)	88%	(82-100%)
Dose intensity combined	Median mg/m^2/day (range)	¥1786	(350*-2600¤)	¥1775	(400*-3000¤)	¥2500	(¥2500-¥2500)	50	(30-60)	50	(30-60)	46	(40-50)
	Dose of standard regimen∫	97	(39-101)	96	(39-101)	99	(69-100)	98%	(60-120%)	98%	(60-120%)	92%	(80-100%)
	Adjusted dose of standard regimenℂ	99	(39-101)	99	(39-101)	100	(69-100)	100%	(62-120%)	100%	(62-120%)	92%	(80-100%)

¥ Capecitabine, * Nordic bolus regimen, ¤ Infused de Gramont; ∫ standard doses presented in S1; ℂ adjusted for age and renal function according to principles in Winther et al., 2019[31]

Supplementary Table S5. Cardiac evaluation and treatment for cardiotoxicity during fluoropyrimidine therapy.

		Fluoropyrimidine causing cardiotoxicity						Switch to S-1 based therapy					
		Total		No recurrent cardiotoxicity		Recurrent cardiotoxicity		Total		No recurrent cardiotoxicity		Recurrent cardiotoxicity	
		200	100 %	192	96 %	8	4 %	200	100 %	192	96 %	8	4 %
**Cardiac evaluation**													
Cardiovascular system	Normal	166	83 %	159	83 %	7	88 %	154	77 %	148	77 %	6	75 %
	Significant abnormalities	18	9 %	17	9 %	1	13 %	9	5 %	7	4 %	2	25 %
	N/A	16	8 %	16	8 %	0	0 %	37	19 %	37	19 %	0	0 %
ECG ʃ	Normal	64	32 %	61	32 %	3	38 %	34	17 %	29	15 %	5	63 %
	Non-significant abnormalities	44	22 %	43	22 %	1	13 %	18	9 %	16	8 %	2	25 %
	Significant abnormalities	72	36 %	68	35 %	4	50 %	10	5 %	9	5 %	1	13 %
	Not done	20	10 %	20	10 %	0	0 %	138	69 %	138	72 %	0	0 %
Echocardiogram	Normal	44	22 %	41	21 %	3	38 %	7	4 %	7	4 %	0	0 %
	Non-significant abnormalities	20	10 %	20	10 %	0	0 %	1	1 %	1	1 %	0	0 %
	Significant abnormalities	10	5 %	10	5 %	0	0 %	1	1 %	1	1 %	0	0 %
	Not done	126	63 %	121	63 %	5	63 %	191	96 %	183	95 %	8	100 %
Troponin ¥	Normal	81	41 %	78	41 %	3	38 %	20	10 %	15	8 %	5	63 %
	Elevated	60	30 %	56	29 %	4	50 %	1	1 %	1	1 %	0	0 %
	Not done	59	30 %	58	30 %	1	13 %	179	90 %	176	92 %	3	38 %
ProBNP ₵	Normal	14	7 %	14	7 %	0	0 %	3	2 %	3	2 %	0	0 %
	Elevated	17	9 %	15	8 %	2	25 %	2	1 %	2	1 %	0	0 %
	Not done	169	85 %	163	85 %	6	75 %	195	98 %	187	97 %	8	100 %
Angiography	Normal	37	19 %	34	18 %	3	38 %	1	1 %	1	1 %	0	0 %
	Non-significant abnormalities	9	5 %	9	5 %	0	0 %	0	0 %	0	0 %	0	0 %
	Significant abnormalities	11	6 %	10	5 %	1	13 %	0	0 %	0	0 %	0	0 %
	Not done	143	72 %	139	72 %	4	50 %	199	100 %	191	100 %	8	100 %
Other*	Normal	29	15 %	28	15 %	1	13 %	1	1 %	0	0 %	1	13 %
	Non-significant abnormalities	11	6 %	8	4 %	3	38 %	2	1 %	1	1 %	1	13 %
	Significant abnormalities	5	3 %	5	3 %	0	0 %	0	0 %	0	0 %	0	0 %
	Not done	155	78 %	151	79 %	4	50 %	197	99 %	191	100 %	6	75 %
**Treatment for cardiotoxicity**													
Any treatment	Yes	100	50 %	95	49 %	5	63 %	15	8 %	13	7 %	2	25 %
	No	100	50 %	97	51 %	3	38 %	185	93 %	181	94 %	6	75 %
Intervention	Yes	9	5 %	8	4 %	1	13 %	0	0 %	0	0 %	0	0 %
	No	191	96 %	184	96 %	7	88 %	200	100 %	192	100 %	8	100 %
Medical therapy	Yes	94	47 %	89	46 %	5	63 %	15	8 %	13	7 %	2	25 %
	No	106	53 %	103	54 %	3	38 %	185	93 %	181	94 %	6	75 %
Cardiovascular medication	C01 Cardiac Therapy	51	26 %	46	24 %	5	63 %	7	4 %	5	3 %	2	25 %
	C03 Diuretics	3	2 %	3	2 %	0	0 %	4	2 %	4	2 %	0	0 %
	C07 Beta Blocking Agents	18	9 %	18	9 %	0	0 %	2	1 %	2	1 %	0	0 %
	C08 Calcium Channel Blockers	12	6 %	12	6 %	0	0 %	1	1 %	1	1 %	0	0 %
	C09 Renin Angiotensin System	8	4 %	8	4 %	0	0 %	3	2 %	3	2 %	0	0 %
	C10 Lipid Modifying Agents	14	7 %	14	7 %	0	0 %	2	1 %	2	1 %	0	0 %
Other medication	A02 Drugs for acid related disorders	11	6 %	11	6 %	0	0 %	2	1 %	2	1 %	0	0 %
	B01 Antithrombotic medication	43	22 %	41	21 %	2	25 %	5	3 %	5	3 %	0	0 %
	N02 Analgesics	26	13 %	24	13 %	2	25 %	1	1 %	1	1 %	0	0 %
	N05 Psycholeptics	7	4 %	5	3 %	2	25 %	0	0 %	0	0 %	0	0 %

* Other such as stress test (n=20+1), CT (n=7), Perfusion scintigraphy (n=5+1), MRI (n=3+1), PET (n=2), Holter (n=2).

ʃ At the initial cardiotoxic event 180 (90%) patients had an ECG and significant abnormalities were found in 72 (30%) cases. Two patients had ECG abnormalities at recurrent cardiotoxic event during S-1, including 1 case of ischaemia and 1 case of premature ventricular contractions. Both had ischaemic ECG at initial cardiotoxic event. Altogether 21 (11%) patients had an ECG during rechallenge including 10 (5%) cases with significant abnormalities.

¥ At the initial cardiotoxic event, 141 (71%) patients had a troponin test and 60 (30%) of them had an elevated troponin level. Only one case of clinically irrelevant elevated troponin level increase was recorded at rechallenge (21 recorded troponin tests).

₵ At the initial cardiotoxic event 31 (18%) patients had proBNP level tested and ProBNP was elevated in 17 (9%) patients. No clinically relevant abnormal proBNP levels were recorded at rechallenge (6 tested).

Supplementary Table S6. Adverse events during treatment with the fluoropyrimidine causing cardiotoxicity and during S-1 based therapy.

		Fluoropyrimidine causing cardiotoxicity						Switch to S-1 based therapy					
		Total		No recurrent cardiotoxicity		Recurrent cardiotoxicity		Total		No recurrent cardiotoxicity		Recurrent cardiotoxicity	
		200	100%	192	96%	8	4%	200	100%	192	96%	8	4%
Non-haematological, grade 2–4													
	Peripheral neuropathy	10	5%	10	5%	0	0%	16	8%	15	8%	1	13%
	Nausea	5	3%	4	2%	1	13%	5	3%	4	2%	1	13%
	Diarrhoea	4	2%	4	2%	0	0%	6	3%	5	3%	1	13%
	Hand-foot syndrome	3	2%	3	2%	0	0%	1	1%	1	1%	0	0%
	Infection	2	1%	2	1%	0	0%	3	2%	3	2%	0	0%
	Stomatitis	1	1%	1	1%	0	0%	0	0%	0	0%	0	0%
	Laryngospasm	1	1%	1	1%	0	0%	0	0%	0	0%	0	0%
	Dyspnoea	1	1%	1	1%	0	0%	0	0%	0	0%	0	0%
	Hypertension	1	1%	1	1%	0	0%	0	0%	0	0%	0	0%
	Thromboembolism	1	1%	1	1%	0	0%	3	2%	3	2%	0	0%
	Epistaxis	1	1%	1	1%	0	0%	0	0%	0	0%	0	0%
	Blood bilirubin increased	0	0%	0	0%	0	0%	1	1%	1	1%	0	0%
	Acute kidney injury	0	0%	0	0%	0	0%	1	1%	1	1%	0	0%
	Abdominal pain	0	0%	0	0%	0	0%	1	1%	1	1%	0	0%
	Trigeminal nerve disorder	0	0%	0	0%	0	0%	1	1%	1	1%	0	0%
	Any	30	15%	29	15%	1	13%	43	22%	40	21%	3	38%
Haematological, grade 3–4													
	Neutropenia	1	1%	1	1%	0	0%	11	6%	10	5%	1	13%
	Leucopenia	0	0%	0	0%	0	0%	1	1%	1	1%	0	0%
	Any	1	1%	1	1%	0	0%	12	6%	11	6%	1	13%

Supplementary Table S7. Patient characteristics of the eight cases with recurrent cardiotoxicity.

Sex	Female	Male	Male	Male	Female	Male	Male	Female
Age	69	58	60	72	70	68	51	58
Diagnosis	Metastatic colon cancer	Rectal cancer	Biliary cancer	Rectal cancer	Metastatic colon cancer	Rectal cancer	Metastatic colon cancer	Metastatic colon cancer
Relevant comorbidities	Panic attacks with palpitations	No	No	Hypertension, myocardial ischemia	Hypertension	Hypertension, myocardial ischemia	No	Hypertension, myocardial ischemia, dilated cardiomyopathy
ECOG	1	1	0	0	0	1	0	1
Regimen	Capecitabine, oxaliplatin, bevacizumab	Capecitabine, oxaliplatin	Capecitabine	Capecitabine, oxaliplatin	Fluorouracil, folinic acid, oxaliplatin	Capecitabine, oxaliplatin	Capecitabine, oxaliplatin, bevacizumab	Capecitabine, oxaliplatin, bevacizumab
Cardiotoxicity	ACS, tachycardia	Cardiac arrest, ACS, tachycardia	Tachycardia	Chest pain	ACS	Chest pain	Chest pain	ACS
Grade	3	4	1	2	3	2	2	3
Cycle of onset	1	1	1	2	1	1	1	14
Days to onset from the start of regimen	11	4	5	27	1	6	4	466
Outcome	Recovered	Recovered with sequelae	Recovered	Recovered	Recovered	Recovered	Recovered	Recovered
Action taken with treatment	Permanently discontinued	Permanently discontinued	Permanently discontinued	Permanently discontinued	Permanently discontinued	Permanently discontinued	Permanently discontinued	Permanently discontinued
Assessed causality	Probably related	Related	Probably related	Probably related	Related	Possibly related	Probably related	Probably related
ECG	Ischemia	Ventricular tachycardia (TdP)	Normal	Normal	Ischemia	Premature atrial contractions	Normal	Ischemia
Troponin	Elevated	Elevated	Not done	Elevated	Elevated	Normal	Normal	Normal
Cardioecho	Normal	Normal	Not done	Not done	Normal	Not done	Not done	Not done
ProBNP	Elevated	Not done	Not done	Not done	Elevated	Not done	Not done	Not done
Angiography	Normal	Normal	Not done	Not done	Normal	Not done	Not done	LAda 60% stenosis
Other	Perfusion scintigraphy, no significant abnormalities	No	Stress test, no significant abnormalities	No	MRI, nonsignificant abnormalities	No	No	No
Treatment of cardiotoxicity	Medical	Medical, DDDR pacemaker	No	Medical	Medical	No	No	Medical
S-1-containing regimen	S-1	S-1, oxaliplatin	S-1	S-1, oxaliplatin	S-1, irinotecan	S-1, oxaliplatin	S-1, oxaliplatin, bevacizumab	S-1, oxaliplatin
Cardiotoxicity	Tachycardia	Tachycardia	Tachycardia	Chest pain	Chest pain	Chest pain	Chest pain	Chest pain
Grade	1	2	2	2	1	1	1	1
Cycle of onset	1	1	1	1	2	3	5	10
Days to onset from the start of regimen	7	10	5	6	22	38	95	195
Outcome	Recovered	Recovered	Recovered	Recovered	Recovered	Recovered	Recovered	Recovered
Action taken with treatment	Dose delayed	Permanently discontinued	Permanently discontinued	Permanently discontinued	Temporarily cancelled	None	None	Dose reduced
Assessed causality	Not related	Probably related	Probably related	Possibly related	Possibly related	Not related	Not related	Possibly related
ECG	Premature ventricular contractions	Tachycardia	Normal	Normal	Normal	Normal	Normal	Ischemia
Troponin	Not done	Normal	Not done	Normal	Normal	Not done	Normal	Normal
ProBNP	Not done	Not done	Not done	Not done	Not done	Not done	Not done	Not done
Echocardiogram	Not done	Not done	Not done	Not done	Not done	Not done	Not done	Not done
Angiography	Not done	Not done	Not done	Not done	Not done	Not done	Not done	Not done
Other	Not done	Not done	Not done	Not done	Not done	Perfusion scintigraphy	Not done	Not done
Treatment of cardiotoxicity	Medical	No	No	Medical	No	No	No	Medical

ACS = acute coronary syndrome; ECG = electrocardiogram; MRI = magnetic resonance imaging; PS = performance status; TdP = torsades de pointes

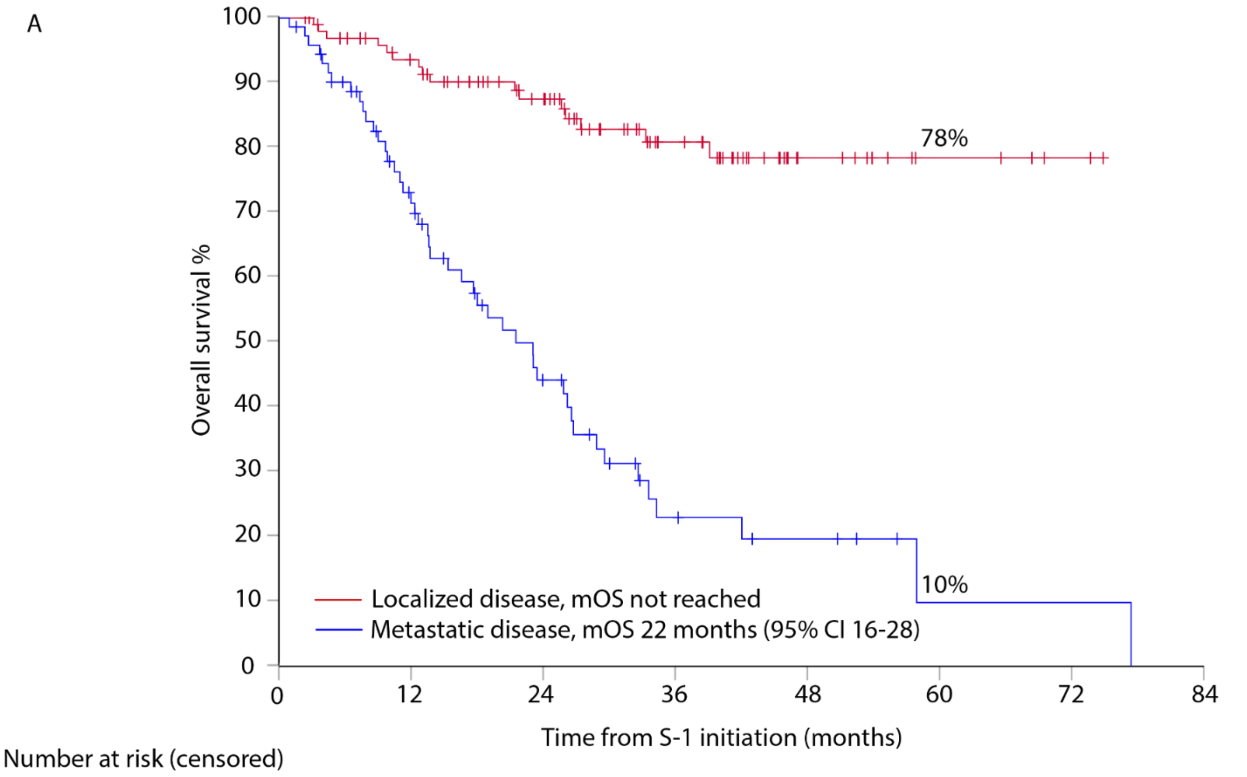

Number at risk (censored)

Localized disease	98 (9)	83 (23)	64 (47)	36 (69)	13 (77)	5 (80)	2 (82)	0 (82)
Metastatic disease	72 (9)	45 (15)	23 (21)	8 (23)	5 (26)	1 (26)	1 (26)	0 (26)

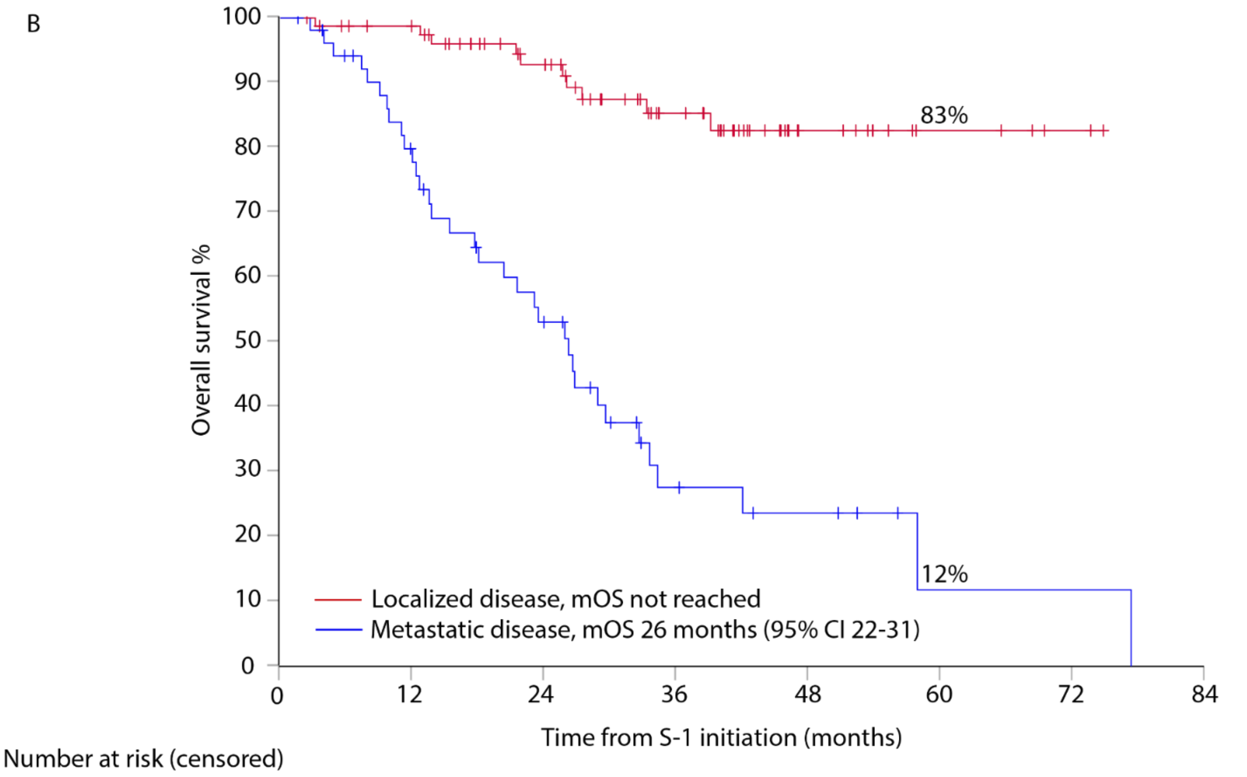

Number at risk (censored)

Localized disease	80 (6)	73 (18)	57 (36)	35 (57)	13 (65)	5 (68)	2 (70)	0 (70)
Metastatic disease	53 (5)	38 (8)	23 (14)	8 (16)	5 (19)	1 (19)	1 (19)	0 (19)

Supplementary Figure S1. Overall survival (OS) from S-1 initiation for patients with localized or metastatic solid cancer (panel A), and colorectal cancer (panel B).

Supplementary Material S1 - Study protocol: Cardioswitch

CARDIOSWITCH

Feasibility of switching fluoropyrimidine due to cardiotoxicity in patients with solid tumours:
A retrospective, international and non-interventional study

Version 1.2, August 14, 2021

Protocol committee:
Pia Österlund, Per Pfeiffer, Halfdan Sørbye, Carl-Henrik Shah, Fridbjörn Sigurdsson, Bengt Glimelius
First draft written by Pia Österlund

National investigators

Dr. Pia Österlund, Assoc. Professor
Dept. of Oncology
Tampere University Hospital
FI-33521 Tampere, Finland
Dr. Per Pfeiffer, Professor
Dept. of Oncology
Odense University Hospital
DK-5000 Odense C, Denmark
Dr. Halfdan Sørbye, Professor
Dept. of Oncology
Haukeland University Hospital
NO-5021 Bergen, Norway
Dr. Carl-Henrik Shah
Dept. of Oncology
Karolinska University Hospital
SE-171 76 Stockholm, Sweden
Dr. Bengt Glimelius
Dept. of Oncology
Uppsala University Hospital
SE-75185 Uppsala, Sweden
Dr. Fridbjörn Sigurdsson
Dept. of Oncology
Landspitali University Hospital
IS-101 Reykjavik, Iceland
Robert-Jan Kwakmann
Dept. of Oncology
AMC Amsterdam
1105 AZ Amsterdam, The Netherlands

Ray McDermott
Dept. of Oncology
Adelaide and Meath Hospital
Dublin, D24 NR04, Ireland

Sponsor

Dr. Pia Österlund
Tampere University Hospital
PO Box 2000, Teiskontie 35
FI-33521 Tampere, Finland

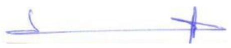

14th August 2021

Signature: ______________________________ **Date:** __________
Principal Investigator: Dr. Pia Österlund, Assoc. Professor

STUDY SUMMARY

**Study title**	Feasibility of switching fluoropyrimidine due to cardiotoxicity in patients with solid tumours: A retrospective, international and non-interventional study<br>**Version 1.2, August 14, 2021**
**Sponsor**	Assoc. Professor Pia Österlund, Department of Oncology, Tampere University Hospital, Finland
**Protocol committee**	Assoc. Professor Pia Österlund, Department of Oncology. Tampere University Hospital<br>Professor Per Pfeiffer, Department of Oncology. Odense University Hospital, Denmark<br>Professor Halfdan Sørbye, Department of Oncology. Haukeland University Hospital, Bergen Norway<br>Oncologist Carl-Henrik Shah, MD, Department of Oncology, Karolinska University Hospital, Sweden<br>Professor Bengt Glimelius, Department of Oncology. Akademiska Hospital, Uppsala University<br>Oncologist Fridbjörn Sigurdsson, MD, Department of Oncology. Landspitali University Hospital, Reykjavik, Iceland.
**Institutions**	All institutions in Europe having had fluoropyrimidine related cardiotoxicity, switch cases with fluoropyrimidines due to cardiotoxicity or prophylactic use of S-1 or another fluoropyrimidine due to cardiac comorbidity.<br>Approximately 30 known cases at the institutions participating in the protocol committee at the time of protocol writing.
**Population**	Patients having perceived cardiotoxicity during a fluorouracil-based treatment for solid tumours, with switch to a different 5-fluorouracil modality or S-1 (Teysuno®), patients with fluoropyridine related cardiotoxicity and prophylactic use of S-1 or another fluoropydimidine due to cardiac comorbicity. Re-challenge with the same fluoropyrimidine.
**Background**	Fluoropyrimidine chemotherapy agents, such as 5-fluorouracil and capecitabine, are occasionally associated with cardiotoxicity that may manifest as chest pain, ECG alterations, cardiac arrhythmia, and rarely myocardial infarction and sudden death. Clinical fluoropyrimidine cardiotoxicity is infrequent (1-8% of patients), but subclinical toxicity may be much more common (up to one third of patients). The underlying mechanisms are not well understood, but they may include abnormal coronary artery contractility or spasm, and myocardial toxicity. Cardiotoxicity may be less frequent with S-1 (a combination of tegafur, gimeracil and oteracil at a molar ratio of 1:0.4:1) as compared with 5-fluorouracil and capecitabine, but head-to-head comparisons are lacking.<br>Anecdotal evidence suggests that patients who have cardiotoxicity on other fluoropyrimidines may be successfully treated with S-1. The purpose of this retrospective study is to compare different 5-fluorouracil-based dosing modalities and S-1, and compare cardiotoxicity during these treatments.<br>The patient population was treated for solid tumours with a 5-fluorouracil based regimen and had a cardiac event grade 1-4. All patients were re-challenged with a different fluoropyrimidine or S-1 and assessed for cardiotoxicity during re-challenge.

**Endpoints**	**Primary**<br>• Cardiac tolerability following cardiotoxicity initiated switch of **fluoropyrimidine to S-1**<br>**Secondary**<br>• Cardiac tolerability following cardiotoxicity initiated switch to any **fluoropyrimidine**<br>• Cardiotoxicity profile during fluoropyrimidine treatment<br>• Cardiotoxicity profile during prophylactic use of S-1, trifluridin/tipiracil or another fluoropyrimidine<br>• Frequency and severity of cardiac symptoms during different fluoropyrimidines and the correlation with other added cytotoxics or biologics<br>• Diagnostic work-up for cardiotoxicity in real world data<br>• Time-lines for cardiotoxicity during fluoropyrimidine treatment<br>• Dose-intensity of the therapy at cardiotoxicity<br>• The alterations of (if evaluated):<br>o ECG changes<br>o Left ventricle regional wall motion abnormalities or global systolic dysfunction<br>o Coronary artery status on angiogram<br>o Cardiac arrhythmias in ECG, Holter or cardiac monitor registration<br>o Plasma troponin concentration and other cardiac enzymes and laboratory tests<br>o Serum alpha-fluoro-beta-alanine (FBAL) concentration
**Safety Criteria for Evaluation**	Standard safety monitoring and grading using National Cancer Institute (NCI) Common Terminology Criteria for Adverse Events (CTCAE) Version 4.0 will be used.
**Methodology**	Multicentre retrospective trial collecting data into a database.
**Sample size**	30-200 patients
**Treatment**	**Cardiotoxicity during a fluoropyrimidine based chemotherapy regimen**<br>• Protracted 5-fluorouracil<br>• 5-fluorouracil combined with leucovorin and given as a bolus regimen, i.e. Mayo, Roswell-Park, Nordic FLv or equivalent<br>• 5-fluorouracil combined with leucovorin and given as an infusional regimen, i.e. de Gramont or equivalent<br>• Peroral fluoropyrimidines such as capecitabine, UFT, carmofur, tegafur, S-1 and equivalent<br>• Other fluoropyrimidines (trifluridine-tiperacil)<br>**Added drugs**<br>• Cytotoxics: Including oxaliplatin, cisplatin, carboplatin, gemcitabine, irinotecan, paclitaxel, docetaxel, epirubicin, doxorubicin and equivalent<br>• Biologics: Including bevacizumab, panitumumab, cetuximab, trastuzumab, tyrosine kinase inhibitors and equivalent

**Inclusion criteria**	• Solid tumours<br>• Cardiotoxicity grade 1-4 during fluoropyrimidine-based treatment<br>• Re-challenge with a different fluoropyrimidine-based therapy
**Exclusion criteria**	• Participation in a trial with experimental drugs
**Statistical analysis**	**Patient Disposition**<br>The number of patients receiving each fluoropyrimidine regimen will be presented as summary statistics.<br>**Patient Baseline Characteristics**<br>Patient characteristics at baseline will be summarized in frequency tables or with summary statistics for continuous variables.<br>**Study Treatment**<br>The drug administration profile will be summarized for different fluoropyrimidines, added cytotoxics and biologics with respect to number of cycles given when cardiotoxicity occurred and at re-challenge, the dose intensity, dose modifications, and reasons for re-challenge.<br>**Safety analyses**<br>The analysis of cardiac toxicity will be carried out in the Safety population. The primary comparison is cardiotoxicity during first fluoropyrimidine based chemotherapy vs. cardiotoxicity including all patients with a cardiotoxicity event on first fluoropyrimidine and cardiotoxicity assessment on second fluoropyrimidine treatment in the analysis and the competing risk 95% confidence interval will be calculated. The primary analysis will be performed on patients switching to S-1 and the secondary comparison will be carried out between the different fluoropyrimidine groups.<br>The safety evaluations will focus on AEs, diagnostic findings and laboratory assessments. All patients included in the Safety population will be evaluated.<br>AEs will be coded according to the Medical Dictionary for Regulatory Activities (MedDRA) terminology and the severity of the toxicities will be graded according to the NCI CTCAE v4.0, where applicable. Concomitant medications will be coded according to the World Health Organization drug dictionary.<br>All AEs will be summarized (incidence) and listed by the System Organ Class, preferred term, toxicity/severity grade, and causal relationship to study medication. In addition, separate summaries of SAEs and Grade 3 or 4 AEs will be presented.<br>Diagnostic findings during cardiotoxicity will be described and summarized, if feasible analysed with non-parametric tests. Laboratory parameters will be graded according to the NCI CTCAE v4.0, where applicable. Absolute values and changes from first fluoropyrimidine to re-challenge will be summarized. In addition, worst severity grade, time to event, and time to resolution will also be summarized.<br>**Primary endpoint**<br>The primary endpoint was recurrent cardiotoxicity after switch to S-1-based treatment. The cumulative incidence with its 95% confidence interval (CI) was calculated in a competing risks analysis, where first onset of recurrent cardiotoxicity was the event of interest and stopping of S-1 without recurrent cardiotoxicity a competing risk. It was specified in advance that a probability of recurrent toxicity below 15% would be considered clinically meaningful, and 15% thus should not be included in the upper boundary for 95% CI. The initial estimate for patient inclusion was 30 patients according to an assessment of what easily could be obtained, and

	since this gave a wide 95% CI of 8.5%-21.5%, an upper limit for patient inclusion was set at 200, judged reasonable after recruitment of more centres, that gave a narrow 95% CI of 12.5%-17.5%.<br>**Determination of Sample Size**<br>No power calculation was performed prospectively. In retrospect with a sample size of 200 patients, the power to reject this null hypothesis with a two-sided alpha of 0.05 would be 80% if the true probability would have been 8% (assuming the power for a test of a proportion approximates that of a cumulative incidence).

TABLE OF CONTENTS

STUDY SUMMARY 17
ABBREVIATIONS 22
DEFINITIONS 22
1. BACKGROUND 23
2. STUDY RATIONALE 25
3. STUDY DESIGN 25
4. STUDY POPULATION AND ENDPOINTS 25
5. NUMBER OF PATIENTS 26
6. DATABASE 26
7. CASE REPORT FORM 26
10. SAFETY 27
11. QUALITY CONTROL 27
14. STATISTICS 28
15. ETHICS 29
16. TIME SCHEDULE 29
17. PUBLICATIONS 30
18. ECONOMY 30
19. REFERENCES 31

ABBREVIATIONS

5-FU	5-fluorouracil
AE	Adverse event
CRF	Case report form
CTCAE	Common Terminology Criteria for Adverse Events
DPD	Dihydropyrimidine dehydrogenase
ECG	Electrocardiogram
FBAL	α-fluoro-β-alanine
ICH	International Council for Harmonisation of Technical Requirements for Pharmaceuticals for Human Use
i.v.	Intravenous
LV	Leucovorin
MedDRA	Medical Dictionary for Regulatory Activities
NCI	National Cancer Institute

DEFINITIONS

S-1	Combination of tegafur, gimeracil and oteracil at a molar ratio of 1:0.4:1 and oteracil at a molar ratio of 1:0.4:1

1. BACKGROUND

Fluoropyrimidines

Fluoropyrimidines (5-fluorouracil (5-FU) derivates) have shown efficacy against a variety of cancers and remain the backbone of systemic treatment in solid tumours. For many decades, 5-FU with or without leucovorin (LV) was the only available treatment for patients. Diarrhoea, mucositis, myelosuppression as neutropenia and hand-foot syndrome are among the major adverse effects of fluoropyrimidines (Cassidy et al., 2002; de Gramont et al., 1997). Cardiotoxicity is a less common but potentially lethal complication of fluoropyrimidines (J. J. Kwakman et al., 2017).

Capecitabine is an orally administered 5-FU prodrug that on a pharmacologic basis simulates the prolonged infusional administration of 5-FU, and has similar or greater clinical efficacy as bolus and infusional 5-FU in combination regimens (Cassidy et al., 2011; Van Cutsem et al., 2000; Van Cutsem et al., 2001). It is rapidly and extensively absorbed as an intact molecule (Miwa et al., 1998; Van Cutsem et al., 2000). Thereafter it is metabolized to 5-FU in three steps. The final step is catalysed by thymidine phosphorylase and takes place in the tumour cells. Therefore, capecitabine potentially offers a reduction of the systemic exposure to 5-FU and maximizes the 5-FU concentration within the tumour tissue.

S-1 (Teysuno®) is third generation oral fluoropyrimidine. S-1 is an oral, fixed-dose combination product comprised of tegafur, a fluoropyrimidine prodrug of 5FU, and 2 modulators of 5FU metabolism, gimeracil and oteracil (Sanford, 2013). S-1 was developed to provide an efficacious and safe oral delivery of 5FU. S-1 provides sustained 5FU plasma concentrations with reduced toxicities due to the addition of the other components of the combination. Tegafur is absorbed in the small intestine and converted to the active 5FU by the CYP2A6 enzyme in the liver. Gimeracil inhibits dihydropyrimidine dehydrogenase (DPD) and thus leads to a prolonged exposure to 5FU. Oteracil potassium inhibits the conversion of 5FU to the active metabolite, fluorouridine monophosphate, in the gut. This inhibition leads to a lower concentration of 5FU in the gut, thereby theoretically reducing gastrointestinal toxicity.

The development of S-1 started in Asia, but because of differences in tumour biology and higher activity of CYP2A6 (thereby more effective conversion of S-1 to 5FU in Caucasian) and toxicity compared to Western population, it is necessary to continue the development of S-1 in Caucasian population separately and determine the optimal dosing of the drug for use in different contexts in monotherapy or in combination (Benson, 2013; Chuah et al., 2011; Hong et al., 2012; Miyamoto, Sakamoto, Yoshida, & Baba, 2014; Muro et al., 2010; Sanford, 2013; Winther et al., 2016; Yamada et al., 2013; Ye, Liu, Ge, Zhou, & Liang, 2014). In a retrospective Danish study (Winther et al., 2016) it was concluded that S-1 may safely substitute capecitabine in combination regimens used in solid tumours, also in elderly patients. The incidence of HFS was very low and no unexpected toxicities were registered.

Fluoropyrimidine-Induced Cardiotoxicity

Cardiotoxicity is an infrequent but potentially lethal complication of fluoropyrimidine administration (J. J. Kwakman et al., 2017; Polk, Vaage-Nilsen, Vistisen, & Nielsen, 2013; Sorrentino, Kim, Foderaro, & Truesdell, 2012). Angina-like chest pain is the predominant clinical presentation, frequently occurring during or following the initial courses of fluoropyrimidine administration. In rare cases myocardial infarction, major arrhythmias, heart failure, cardiogenic shock, sudden death, and pericarditis have been reported.

The reported incidence of cardiotoxicity due to 5-FU varies in the literature ranging from 1-68% of patients (J. J. Kwakman et al., 2017; Polk et al., 2013; Sorrentino et al., 2012). High doses and continuous infusion schedules of 5-FU have been associated with higher rates of cardiotoxicity than intravenous (i.v.) bolus regimens. Capecitabine is also associated with a non-negligible incidence rate (3-9%) of cardiotoxicity (Polk et al., 2013).

Only 18% of the patients who develop cardiotoxicity have a history of underlying cardiac disease. Most cases occur in patients without previous coronary disease, and previous heart disease is not predictive for fluoropyrimidine-related cardiotoxicity, but might worsen the cardiotoxicity (Yeh & Bickford, 2009). The overall reported mortality of 5-FU or capecitabine-associated cardiotoxicity in prospective studies varies between 0% and 2.2% (Polk et al., 2013).

The pathophysiology of 5-FU-induced cardiotoxicity has not been completely elucidated. One hypothesis attributes 5-FU cardiotoxicity to coronary vasospasm induced by 5-FU or its metabolites (Sorrentino et al., 2012). Coronary vasospasm has been demonstrated in animal models as well as in human vascular studies during 5-FU infusion. However, some characteristics of 5-FU-related cardiotoxicity are inconsistent with ischemic coronary heart disease. Reintroduction of 5-FU in patients with a previous adverse cardiac event did not result in coronary spasm as evidenced by coronary angiography, and no vasospasms were documented angiographically during symptomatic attacks. Moreover, in many cases vasodilator drugs cannot prevent cardiotoxicity after re-challenge with 5-FU. (Burger & Mannino, 1987; Deboever, Hiltrop, Cool, & Lambrecht, 2013; Kosmas et al., 2008; Saif, Syrigos, & Katirtzoglou, 2009)

Besides coronary spasms other mechanisms probably contribute to cardiotoxicity. 5-FU therapy induces global reversible endothelial injury, with impaired vasodilatation of the vascular smooth muscle (and vasoconstriction), leading to a procoagulant state (Sorrentino et al., 2012). The degradation products of 5-FU catabolism are probably involved in a direct toxicity on the myocardium inducing a toxic cardiomyopathic picture. In animal studies, rabbits receiving a single high dose bolus injection of 5-FU suffered massive haemorrhagic myocardial infarction with evidence of proximal coronary vasospasm. Animals in the repeated lower dose infusion group however demonstrated histologic changes mimicking toxic myocarditis, with left ventricular hypertrophy, thickening of intramyocardial arteries, and disseminated apoptosis in myocardial cells of the epicardium and endothelial cells of the distal coronary artery. In patients with 5-FU cardiotoxicity, echocardiography revealed a decreased ejection fraction and a significant global or regional left ventricular dysfunction consistent with a cardiomyopathic picture. By monitoring plasma levels of N-terminal pro-brain natriuretic peptide, it was recently documented that not only patients with clinically symptomatic cardiac events but also most of the patients treated with 5-FU have reversible clinical cardiac (Deboever et al., 2013).
Eighty-five percent to 90% of the i.v. administered 5-FU is rapidly catabolized (mainly in the liver) by DPD to 5, 6 dihydrofluorouracil and further to alfa-fluoro-beta-ureidopropionic acid and α-fluoro-β-alanine (FBAL). Approximately 90% of 5-FU in plasma is excreted as FBAL in the urine within 24 hours. FBAL is further catabolized into fluoroacetate and F-citrate. F-citrate inhibits the Krebs cycle by inhibition of aconitase, leading to a build-up of citrate and limiting the cellular production of adenosine triphosphate. Fluoroacetate is known to be highly cardiotoxic and neurotoxic. In animal experiments with open-chest guinea pigs exposed to 5-FU accumulation of citrate within the myocardium was documented, indeed suggesting a malfunction of the Krebs cycle by inhibition of aconitase by F-citrate. Individual susceptibility to cardiotoxicity might result from genetic variations in the activity of the enzyme pathways involved in the catabolism of 5-FU, leading to variable levels of the cardiotoxic degradation products. (Deboever et al., 2013)
Recent clinical research programs have been focused on the potential of oral prodrugs to replace 5-FU or capecitabine with the aim of obtaining better efficacy, but also a better toxicity profile, and overall better tolerance.

S-1 and cardiac safety

Due to the influence of Gimeracil on the DPD catabolism and the low dose of Tegafur in the compound compared to other fluoropyrimidines, the risk of cardiotoxicity related to S-1 could be lower than that of i.v. 5-FU or capecitabine.
One Phase I study performed in Europe has evaluated cardiotoxicity of S-1. The results of the study were published at the 2013 American Association for Cancer Research congress. The objective of the study was to investigate the effect of S-1 on cardiac repolarization evaluated during S-1 treatment under controlled conditions with assessment of 24-hour (h) Holter monitoring electrocardiograms (ECGs). 56 patients with advanced solid tumours were enrolled, and the cardiac safety results could be analysed for 49 patients. The patients received S-1 30mg/m^2 BID from D1 to Day 14 q3w. This trial investigated the effect of S-1 vs the baseline and compared to placebo with cardiac repolarization as the endpoint (QTc; the corrected QT interval). During cycle 1, the patients were assessed with 24-h Holter monitoring ECGs after each dose of S-1.
The results were reported as followed:

- The 1-sided 95% upper confidence boundaries for the QTc changes were completely within the 10 msec boundary set by the ICH guideline.
- The mean changes relative to placebo (as adjusted for the baseline measurements) following single-doses of S-1 did not indicate any effect on the QTc.
- Following multiple doses of S-1, all mean increases in the QTc interval compared to placebo were below the threshold of (regulatory) concern.

Forty-two (75.0%) patients reported 225 adverse events (AEs). Only 5 patients (8.9%) experienced treatment-related Grade 3 or higher events, which included anaemia, neutropenia and diarrhoea. One patient discontinued treatment due to grade 2 hand-foot syndrome. No cardiac serious AEs or treatment-related cardiac AEs were observed. No cases of syncope or arrhythmia were reported (Grade 1 atrial fibrillation, 1 patient; Grade1 tachycardia, 1 patient; both unrelated to treatment). S-1 was considered well-tolerated in these heavily treated patients with no unexpected treatment-related AEs that resulted in death or AEs leading to discontinuation of the treatment occurred. No effect on cardiac repolarization was thus observed among the 49 patients analysed.
The good cardiac safety profile of S-1 is also supported by a survey performed in Japan on nearly 4,000 patients treated with S-1 (Nagashima, Ohtsu, Yoshida, & Ito, 2005). No more than 0.2% all grades and 0.05% Grade 3-4 cardiac events were observed, which figures compare favourably with the rates reported for this class of drugs.

A case-report from the Netherlands presented seven cases with vasospasm during capecitabine based chemotherapy and none had recurrence on rechallenge with S-1 based as single agent or in combination (J. J. M. Kwakman et al., 2017). This is in line with previous findings and S-1 might thus be safer as compared to other fluoropyrimidines in terms of cardiotoxicity.
The ongoing randomized phase II trial FLUheart compares cardiotoxicity during capecitabine and S-1 based chemotherapy in combination with oxaliplatin as treatment for gastrointestinal adenocarcinoma.

2. STUDY RATIONALE

Fluoropyrimidine chemotherapy agents, such as 5-fluorouracil and capecitabine, are occasionally associated with cardiotoxicity that may manifest as chest pain, ECG alterations, cardiac arrhythmia, and rarely myocardial infarction, heart failure, sudden death and other cardiac adverse events. Clinical fluoropyrimidine cardiotoxicity is infrequent (1-8% of patients), but subclinical toxicity may be much more common (up to one third of patients). The underlying mechanisms are not well understood, but they may include abnormal coronary artery contractility or spasm, and myocardial toxicity.
Cardiotoxicity may be less frequent with S-1 (a combination of tegafur, gimeracil and oteracil) as compared with 5-fluorouracil and capecitabine, but head-to-head comparisons and real-world data are scarce. Anecdotal evidence suggests that patients who have chest/cardiac pain on other fluoropyrimidines may be successfully treated with S-1. A small systematic patient-series have been collected previously (J. J. M. Kwakman et al., 2017).
The purpose of the present study is to evaluate cardiotoxicity during re-challenge of a different modality of fluoropyrimidine (primary end-point S-1 and secondary any other fluoropyrimidine) after having perceived cardiotoxicity with a fluoropyrimidine based regimen previously. The patient population is being treated for solid tumours.

3. STUDY DESIGN

This is the assessment of a specific evaluation of cardiac safety for patients with solid tumours who have experienced cardiotoxicity grade 1-4 during treatment with a fluoropyrimidine based treatment of which some are re-challenged with a different fluoropyrimidine.
This multicentre, retrospective database is built to assess the impact on the cardiac and global safety of two different fluoropyrimidine based treatment regimens, of which the first has caused cardiotoxicity grade 1-4 (Figure 1).
Cardiac data will be collected by medical record review from initiation of first fluoropyrimidine-based treatment and switch to second fluoropyrimidine-based treatment until death or last follow-up. Basic demographics, cancer and treatment information from the whole course of cancer until death or last follow-up.

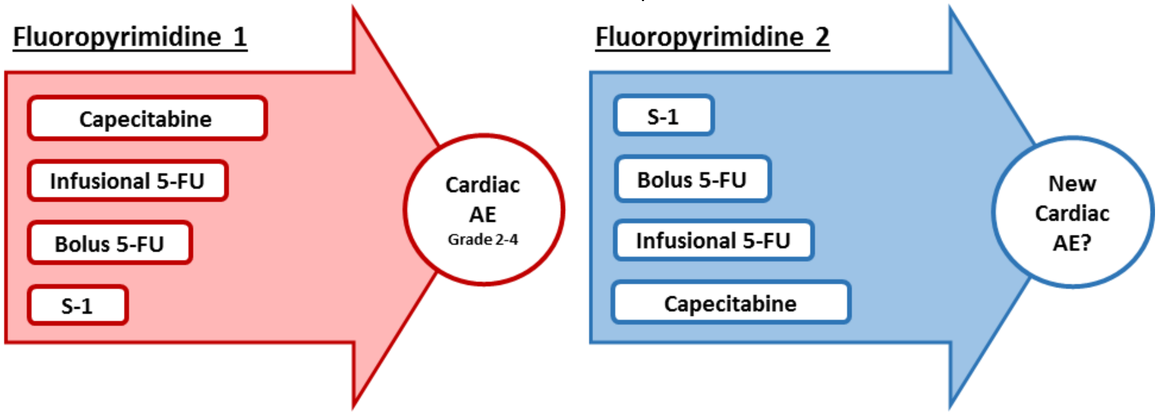

Figure 1. Overall study design

4. STUDY POPULATION AND ENDPOINTS

All consecutive patients who fulfil the following inclusion criteria will be included in the database until the target number of patients has been included:

- Solid tumours
- Cardiotoxicity grade 1-4 during fluoropyrimidine-based treatment
- Re-challenge with the same or a different fluoropyrimidine-based treatment. Primary endpoint is switch to S-1 and secondary any fluoropyrimidine population.
- Prophylactic use of S-1 or another fluoropyrimidine due to cardiac comorbicity.

Primary endpoint

- Cardiac tolerability following cardiotoxicity initiated switch of **fluoropyrimidine to S-1**

Secondary

- Cardiac tolerability following cardiotoxicity initiated switch to any **fluoropyrimidine**
- Cardiotoxicity profile during fluoropyrimidine treatment
- Cardiotoxicity profile during prophylactic use of S-1, trifluridin/tipiracil or another fluoropyrimidine
- Frequency and severity of cardiac symptoms during different fluoropyrimidines and the correlation with other added cytotoxics or biologics
- Diagnostic work-up for cardiotoxicity in real world data
- Time-lines for cardiotoxicity during fluoropyrimidine treatment
- Dose-intensity of the therapy at cardiotoxicity
- The alterations of (if evaluated):
    - ECG changes
    - Left ventricle regional wall motion abnormalities or global systolic dysfunction
    - Coronary artery status on angiogram
    - Cardiac arrhythmias in ECG, Holter or cardiac monitor registration
    - Plasma troponin concentration and other cardiac enzymes and laboratory tests
    - Serum alpha-fluoro-beta-alanine (FBAL) concentration

5. NUMBER OF PATIENTS

30-200 patients in total are expected to be included at the institutions participating in the study in the Nordic countries. The aim is to also include other European countries depending on local regulations regarding retrospective patient registries.
Eligible population: Patients who had a grade 1-4 cardiac event during fluoropyrimidine treatment of which some were re-challenged with a different fluoropyrimidine-based chemotherapy, or had S-1 or another fluoropydimidine prophylactically.

6. DATABASE

An electronic CRF will be completed for each patient included in the study. The Investigator is responsible for completing the CRFs using source data (medical records and chemotherapy administration records) and for ensuring the accuracy, completeness, legibility and timeliness of the data recorded in the CRFs. Data reported in the CRF that are derived from source documents should be consistent with the source documents. Any discrepancies should be explained.
Blank electronic CRFs will be provided by the Sponsor. Each patient will be electronically assigned a patient identifier comprising the Investigator's name and a 6-digit number. At each study centre, a paper list of identifiers for patients at that centre will be created and maintained locally. The handling of data by the Sponsor after receipt of the CRFs may generate additional requests to which the Investigator is obliged to respond by confirming or modifying the data in question.
A certified database has been created by Raisoft Ltd (www.raisoft.com).

7. CASE REPORT FORM

The following information will be recorded in the CRFs:
A) Baseline status and lifestyle
B) Co-Morbidities
C) Concomitant medications
D) Tumour characteristics
E) Cancer treatments prior to the first fluoropyrimidine regimen (previous regimens)
F) Fluoropyrimidine regimen causing cardiotoxicity (including number of cycles, dose modifications)
G) Fluoropyrimidine regimen after cardiotoxicity (including number of cycles, dose modifications)
H) Information about cardiac AE, diagnostics, treatment and outcome
I) Other hematological AEs that are grade 2-4 and hematologic grade 3-4 are collected. DPD status if available
J) Follow-up information for DFS or PFS and OS calculation.

10. SAFETY

Recording of cardiac adverse events

All cardiac AEs, i.e. cardiac events during fluoropyrimidine 1 and after switching to fluoropyrimidine 2, must be documented in the appropriate section of the CRF.
The following aspects must be recorded for each event in the CRF:

- A description of the AE in medical terms, not as reported by the subject
- Date of onset (start date)
- Date of recovery (stop date), if available
- Action taken regarding treatment
- Grade, as assessed retrospectively by the Investigator according to the definitions in CTCAE version 4.0:
    - 1 = mild
    - 2 = moderate
    - 3 = severe
    - 4 = life-threatening or disabling
    - 5 = death related to AE
- Causal relationship to fluoropyrimidine treatment, as assessed retrospectively by the Investigator:

Not related
An AE with an incompatible time relationship to fluoropyrimidine treatment and which can be explained by underlying disease or other drugs or is incontrovertibly not related to fluoropyrimidine treatment.
Possibly related
An AE with a reasonable time relationship to fluoropyrimidine treatment, but which could also be explained by concurrent disease or other medications.
Probably related
An AE with a reasonable time relationship to fluoropyrimidine treatment and which is unlikely to be attributed to concurrent disease or other medications.
Related
An AE with a plausible time relationship to fluoropyrimidine treatment and which cannot be explained by concurrent disease or concomitant medications.

Safety evaluations

The safety evaluations will focus on AEs judged to be related to fluoropyrimidine treatment, diagnostic findings and laboratory assessments relevant to cardiotoxicity. All patients included will be evaluated in the safety analysis. All cardiac AEs will be summarized, and in addition separate summaries of AEs will be presented.

11. QUALITY CONTROL

Investigator

The Investigator agrees to conduct the study in accordance with the Clinical Trial Protocol, applicable regulatory requirements and local legislation covering data protection. The Investigator is required to ensure compliance with the protocol and other procedures provided by the Sponsor. For the purpose of ensuring compliance with the protocol, applicable regulatory requirements and local legislation covering data protection, the Investigator will permit auditing by the Sponsor or representatives and inspections by regulatory authorities.
The Investigator shall take appropriate measures required by the Sponsor to take corrective actions for all problems found during audits and or inspections.
Personnel involved in conducting this study will be qualified by education, training and experience to perform their respective tasks.

Source Documentation

At a minimum, source documentation/essential documents (medical records and chemotherapy administration records) must be available for the following to confirm data collected in the CRF: subject identification, eligibility, study identification and safety parameters, including AEs. To enable audits and evaluations by the sponsor and inspections by regulatory authorities, the Investigator shall keep records/essential documents of the trial for 10 years.

14. STATISTICS

Patient Baseline Characteristics

Patient characteristics at baseline will be summarized in frequency tables or with summary statistics for continuous variables.

Fluoropyrimidine Treatments

The drug administration profile will be presented as summary statistics for the different fluoropyrimidines, added cytotoxics and biologics with respect to number of cycles given when cardiotoxicity occurred during fluoropyrimidine 1 (all patients) and during fluoropyrimidine 2 (patients in whom cardiotoxicity occurred), dose intensity, dose modifications and re-challenge. The primary end point is analysed in the switch to S-1 population and secondary in the any fluoropyrimidine population.

Safety Analyses

The primary endpoint is cardiac tolerability following cardiotoxicity initiated switch of **fluoropyrimidine to S-1. The** incidence and type of grade 1-5 cardiotoxicities during the switch to S-1 (primary endpoint) or any fluoropyrimidine (secondary endpoint), (fluoropyrimidine 2, figure 1) and it will be analysed calculated with competing risk 95% confidence interval and using a nonparametric test if appropriate. A secondary analysis will compare the different fluoropyrimidine 1 treatments in terms of cardiotoxicities after switching to fluoropyrimidine 2. Safety in the subpopulation with rechallenge of same fluoropyrimidine and prophylactic use of S.1 or another fluoropyrimidine will be analysed.

The safety evaluations will focus on cardiac AEs, diagnostic findings and laboratory assessments. All patients included in the Safety population will be evaluated.

Cardiac AEs will be coded according to Medical Dictionary for Regulatory Activities (MedDRA) terminology and the severity of the toxicities will be graded according to the NCI CTCAE v4.0, where possible. Concomitant medications will be coded according to the World Health Organization drug dictionary. AEs will be grouped according to System Organ Class, preferred term, worst severity grade, causal relationship to study medication and action taken regarding treatment. Time to event and time to resolution will be presented as summary statistics.

Diagnostic findings during cardiotoxicity will be presented as summary statistics. If feasible, the data will be analysed with non-parametric tests. Laboratory values that are (1) outside the normal range and (2) judged by the Investigator to be clinically significant will be recorded as AEs in the CRFs and graded according to the NCI CTCAE v4.0 and presented as absolute values and changes.

Primary endpoint

The primary endpoint was recurrent cardiotoxicity after switch to S-1-based treatment. The cumulative incidence with its 95% confidence interval (CI) was calculated in a competing risks analysis, where first onset of recurrent cardiotoxicity was the event of interest and stopping of S-1 without recurrent cardiotoxicity a competing risk. It was specified in advance that a probability of recurrent toxicity below 15% would be considered clinically meaningful, and 15% thus should not be included in the upper boundary for 95% CI. The initial estimate for patient inclusion was 30 patients according to an assessment of what easily could be obtained, and since this gave a wide 95% CI of 8.5%-21.5%, an upper limit for patient inclusion was set at 200, judged reasonable after recruitment of more centres, that gave a narrow 95% CI of 12.5%-17.5%.

Sample size

No power calculation has been performed prospectively. Clinical cardiac toxicity is seen in 1-8% of patients receiving fluoropyrimidines, but number of patients that switch due to cardiac toxicity is not known. The number of patients (30-200) has been estimated based on the number of patients expected to meet the inclusion criteria during the study period. After inclusion of 20-30 patients an interim analysis of cardiotoxicity after switching to a second fluoropyrimidine-based regimen will be performed. If the quality of the data is sufficient i.e. feasibility of a fluoropyrimidine switch can be evaluated, inclusion will be continued with the intention of including 100-200 patients in the database. Inclusion of patients may continue during the interim analysis. Inclusion will continue until 31st December 2025, if necessary to include a sufficient number of patients.
In retrospect with a sample size of 200 patients, the power to reject this null hypothesis with a two-sided alpha of 0.05 would be 80% if the true probability would have been 8% (assuming the power for a test of a proportion approximates that of a cumulative incidence).

15. ETHICS

This retrospective study will be conducted in compliance with the protocol and in accordance with the ethical principles put forward in the second Declaration of Helsinki. This study will not affect the treatment of patients who have had cardiotoxicity from a fluoropyrimidine. This study may on the other hand create data on handling patients with a cardiac AE from a fluoropyrimidine and give insights into switch to a different fluoropyrimidine, more than the anecdotal evidence among oncologists and in the literature. No informed consent is required in the Nordic countries entering patient data. In a retrospective patient series informed consent is too difficult to obtain, due to the high mortality of solid tumours. No patient identifier will be included in the database, only the investigators name and a number. Ethics committee approval will be obtained according to local regulation in the participating countries.
Research data will be handled according to the GDPR regulation of the EU. The legal basis for the processing of personal data is public interest as regards public health and scientific research, such as ensuring high standards of quality and safety in healthcare, medicinal products of medical devices according to article 6 (1) (e), 9 (2) (i) and) (2) (j).
The pseudonymized data is collected into the database cardiotox.raisoft.net and are identifiable only by the local investigator at the site and the registry permission holder. The database platform is provided by Raisoft Ltd, which has been security checked by Istekki. The database is secured by log, username, password, registration of use, and access control. Only members of the research group will be given access rights.
Data in the information systems will be coded before statistical analysis. Coded data without sensitive information about identification of subjects can be shared between research groups. Research data will be handled confidentially. Group data analyses will be done coded at which time a single subject cannot be identified without the code. Local code keys will be stored by the principal investigator at each site. The code key will not be given to anyone except research team members of the local site. All gathered data will be handled coded and a single case cannot be identified when data is shared, reported or published. After the study is finished the data will be stored in the datasecure Lokero at Prikanmaan sairaanhoitopiiri for 15 years, after which the data will be destroyed.

16. TIME SCHEDULE

The trial is expected to start soon after ethics committee approval of the protocol has been granted in June 1, 2018. New patients may be added until December 2025.

17. PUBLICATIONS

A study report in abstract form will be prepared after the interim analysis and at end of the study. Moreover, we plan to publish the study in international peer-reviewed journals. The study will be published once it is completed and the final analysis has been performed. Any publication based on the data from this study proceeds from the investigator group, with specification of the participating clinics and responsible contacts. The names on the author list will be given according to the active participation in the design of the protocol, in the identification of eligible and evaluable patients, in the compilation of results and in the production of the article.

The protocol committee writes the first draft. The manuscript will be completed and submitted by the Sponsor-Investigator who will also decide who will be the first author. Co-authors are from those centres that have included most patients, if all cannot be included.

18. ECONOMY

None of the study personnel have any economic involvement in this study. No compensation will be provided for the Investigators, or patients. Nordic Drugs has provided economic support to Raisoft Ltd for the setting up and maintenance of the database and a grant for the study coordinator.

19. REFERENCES

Benson, A. B., 3rd. (2013). S-1: another oral agent for patients with colorectal cancer. *Lancet Oncol, 14*(13), 1244-1245. doi: 10.1016/s1470-2045(13)70533-3

Burger, A. J., & Mannino, S. (1987). 5-Fluorouracil-induced coronary vasospasm. *Am Heart J, 114*(2), 433-436.

Cassidy, J., Clarke, S., Diaz-Rubio, E., Scheithauer, W., Figer, A., Wong, R., . . . Saltz, L. (2011). XELOX vs FOLFOX-4 as first-line therapy for metastatic colorectal cancer: NO16966 updated results. *Br J Cancer, 105*(1), 58-64. doi: 10.1038/bjc.2011.201

Cassidy, J., Twelves, C., Van Cutsem, E., Hoff, P., Bajetta, E., Boyer, M., . . . Capecitabine Colorectal Cancer Study, Group. (2002). First-line oral capecitabine therapy in metastatic colorectal cancer: a favorable safety profile compared with intravenous 5-fluorouracil/leucovorin. *Ann Oncol, 13*(4), 566-575.

Chuah, B., Goh, B. C., Lee, S. C., Soong, R., Lau, F., Mulay, M., . . . Rosen, L. S. (2011). Comparison of the pharmacokinetics and pharmacodynamics of S-1 between Caucasian and East Asian patients. *Cancer Sci, 102*(2), 478-483. doi: 10.1111/j.1349-7006.2010.01793.x

de Gramont, A., Bosset, J. F., Milan, C., Rougier, P., Bouche, O., Etienne, P. L., . . . Bedenne, L. (1997). Randomized trial comparing monthly low-dose leucovorin and fluorouracil bolus with bimonthly high-dose leucovorin and fluorouracil bolus plus continuous infusion for advanced colorectal cancer: a French intergroup study. *J Clin Oncol, 15*(2), 808-815.

Deboever, G., Hiltrop, N., Cool, M., & Lambrecht, G. (2013). Alternative treatment options in colorectal cancer patients with 5-fluorouracil- or capecitabine-induced cardiotoxicity. *Clin Colorectal Cancer, 12*(1), 8-14. doi: 10.1016/j.clcc.2012.09.003

Hong, Y. S., Park, Y. S., Lim, H. Y., Lee, J., Kim, T. W., Kim, K. P., . . . Lee, J. W. (2012). S-1 plus oxaliplatin versus capecitabine plus oxaliplatin for first-line treatment of patients with metastatic colorectal cancer: a randomised, non-inferiority phase 3 trial. *Lancet Oncol, 13*(11), 1125-1132. doi: 10.1016/s1470-2045(12)70363-7

Kosmas, C., Kallistratos, M. S., Kopterides, P., Syrios, J., Skopelitis, H., Mylonakis, N., . . . Tsavaris, N. (2008). Cardiotoxicity of fluoropyrimidines in different schedules of administration: a prospective study. *J Cancer Res Clin Oncol, 134*(1), 75-82. doi: 10.1007/s00432-007-0250-9

Kwakman, J. J. M., Baars, A., van Zweeden, A. A., de Mol, P., Koopman, M., Kok, W. E. M., & Punt, C. J. A. (2017). Case series of patients treated with the oral fluoropyrimidine S-1 after capecitabine-induced coronary artery vasospasm. *Eur J Cancer, 81*, 130-134. doi: 10.1016/j.ejca.2017.05.022

Kwakman, J. J., Simkens, L. H., Mol, L., Kok, W. E., Koopman, M., & Punt, C. J. (2017). Incidence of capecitabine-related cardiotoxicity in different treatment schedules of metastatic colorectal cancer: A retrospective analysis of the CAIRO studies of the Dutch Colorectal Cancer Group. *Eur J Cancer, 76*, 93-99. doi: 10.1016/j.ejca.2017.02.009

Miwa, M., Ura, M., Nishida, M., Sawada, N., Ishikawa, T., Mori, K., . . . Ishitsuka, H. (1998). Design of a novel oral fluoropyrimidine carbamate, capecitabine, which generates 5-fluorouracil selectively in tumours by enzymes concentrated in human liver and cancer tissue. *Eur J Cancer, 34*(8), 1274-1281.

Miyamoto, Y., Sakamoto, Y., Yoshida, N., & Baba, H. (2014). Efficacy of S-1 in colorectal cancer. *Expert Opin Pharmacother, 15*(12), 1761-1770. doi: 10.1517/14656566.2014.937706

Muro, K., Boku, N., Shimada, Y., Tsuji, A., Sameshima, S., Baba, H., . . . Sugihara, K. (2010). Irinotecan plus S-1 (IRIS) versus fluorouracil and folinic acid plus irinotecan (FOLFIRI) as second-line chemotherapy for metastatic colorectal cancer: a randomised phase 2/3 non-inferiority study (FIRIS study). *Lancet Oncol, 11*(9), 853-860. doi: 10.1016/s1470-2045(10)70181-9

Nagashima, F., Ohtsu, A., Yoshida, S., & Ito, K. (2005). Japanese nationwide post-marketing survey of S-1 in patients with advanced gastric cancer. *Gastric Cancer, 8*(1), 6-11. doi: 10.1007/s10120-004-0306-3

Polk, A., Vaage-Nilsen, M., Vistisen, K., & Nielsen, D. L. (2013). Cardiotoxicity in cancer patients treated with 5-fluorouracil or capecitabine: a systematic review of incidence, manifestations and predisposing factors. *Cancer Treat Rev, 39*(8), 974-984. doi: 10.1016/j.ctrv.2013.03.005

Saif, M. W., Syrigos, K. N., & Katirtzoglou, N. A. (2009). S-1: a promising new oral fluoropyrimidine derivative. *Expert Opin Investig Drugs, 18*(3), 335-348. doi: 10.1517/13543780902729412

Sanford, M. (2013). S-1 (Teysuno(R)): a review of its use in advanced gastric cancer in non-Asian populations. *Drugs, 73*(8), 845-855. doi: 10.1007/s40265-013-0062-y

Sorrentino, M. F., Kim, J., Foderaro, A. E., & Truesdell, A. G. (2012). 5-fluorouracil induced cardiotoxicity: review of the literature. *Cardiol J, 19*(5), 453-458.

Van Cutsem, E., Findlay, M., Osterwalder, B., Kocha, W., Dalley, D., Pazdur, R., . . . Verweij, J. (2000). Capecitabine, an oral fluoropyrimidine carbamate with substantial activity in advanced colorectal cancer: results of a randomized phase II study. *J Clin Oncol, 18*(6), 1337-1345.

Van Cutsem, E., Twelves, C., Cassidy, J., Allman, D., Bajetta, E., Boyer, M., . . . Xeloda Colorectal Cancer Study, Group. (2001). Oral capecitabine compared with intravenous fluorouracil plus leucovorin in patients with metastatic colorectal cancer: results of a large phase III study. *J Clin Oncol, 19*(21), 4097-4106.

Winther, S. B., Zubcevic, K., Qvortrup, C., Vestermark, L. W., Jensen, H. A., Krogh, M., . . . Pfeiffer, P. (2016). Experience with S-1 in older Caucasian patients with metastatic colorectal cancer (mCRC): Findings from an observational chart review. *Acta Oncol, 55*(7), 881-885. doi: 10.3109/0284186x.2016.1161825

Yamada, Y., Takahari, D., Matsumoto, H., Baba, H., Nakamura, M., Yoshida, K., . . . Sugihara, K. (2013). Leucovorin, fluorouracil, and oxaliplatin plus bevacizumab versus S-1 and oxaliplatin plus bevacizumab in patients with metastatic colorectal cancer (SOFT): an open-label, non-inferiority, randomised phase 3 trial. *Lancet Oncol, 14*(13), 1278-1286. doi: 10.1016/s1470-2045(13)70490-x

Ye, J. X., Liu, A. Q., Ge, L. Y., Zhou, S. Z., & Liang, Z. G. (2014). Effectiveness and safety profile of S-1-based chemotherapy compared with capecitabine-based chemotherapy for advanced gastric and colorectal cancer: A meta-analysis. *Exp Ther Med, 7*(5), 1271-1278. doi: 10.3892/etm.2014.1576

Yeh, E. T., & Bickford, C. L. (2009). Cardiovascular complications of cancer therapy: incidence, pathogenesis, diagnosis, and management. *J Am Coll Cardiol, 53*(24), 2231-2247. doi: 10.1016/j.jacc.2009.02.050
